# Molecular Mechanism of SARS-CoVs Orf6 Targeting the Rae1–Nup98 Complex to Compete With mRNA Nuclear Export

**DOI:** 10.3389/fmolb.2021.813248

**Published:** 2022-01-12

**Authors:** Tinghan Li, Yibo Wen, Hangtian Guo, Tingting Yang, Haitao Yang, Xiaoyun Ji

**Affiliations:** ^1^ The State Key Laboratory of Pharmaceutical Biotechnology, School of Life Sciences, Institute of Viruses and Infectious Diseases, Chemistry and Biomedicine Innovation Center (ChemBIC), Institute of Artificial Intelligence Biomedicine, Nanjing University, Nanjing, China; ^2^ School of Life Sciences, Tianjin University, Tianjin, China; ^3^ Shanghai Institute for Advanced Immunochemical Studies and School of Life Science and Technology, ShanghaiTech University, Shanghai, China; ^4^ Tianjin International Joint Academy of Biotechnology and Medicine, Tianjin, China; ^5^ Shanghai Clinical Research and Trial Center, Shanghai, China; ^6^ Engineering Research Center of Protein and Peptide Medicine, Ministry of Education, Nanjing, China

**Keywords:** SARS-CoV-2, ORF6, Rae1-Nup98 complex, mRNA nuclear export, crystal structure

## Abstract

The accessory protein Orf6 is uniquely expressed in sarbecoviruses including severe acute respiratory syndrome coronavirus 2 (SARS-CoV-2) which is an ongoing pandemic. SARS-CoV-2 Orf6 antagonizes host interferon signaling by inhibition of mRNA nuclear export through its interactions with the ribonucleic acid export 1 (Rae1)–nucleoporin 98 (Nup98) complex. Here, we confirmed the direct tight binding of Orf6 to the Rae1-Nup98 complex, which competitively inhibits RNA binding. We determined the crystal structures of both SARS-CoV-2 and SARS-CoV-1 Orf6 C-termini in complex with the Rae1–Nup98 heterodimer. In each structure, SARS-CoV Orf6 occupies the same potential mRNA-binding groove of the Rae1–Nup98 complex, comparable to the previously reported structures of other viral proteins complexed with Rae1-Nup98, indicating that the Rae1–Nup98 complex is a common target for different viruses to impair the nuclear export pathway. Structural analysis and biochemical studies highlight the critical role of the highly conserved methionine (M58) of SARS-CoVs Orf6. Altogether our data unravel a mechanistic understanding of SARS-CoVs Orf6 targeting the mRNA-binding site of the Rae1–Nup98 complex to compete with the nuclear export of host mRNA, which further emphasizes that Orf6 is a critical virulence factor of SARS-CoVs.

## Introduction

Coronavirus disease 2019 (COVID-19) (Berlin et al., 2020; Zhu et al., 2020), which is caused by severe acute respiratory syndrome coronavirus 2 (SARS-CoV-2) (Zhang and Holmes, 2020), has brought global pandemic since March 2020. As of 2 December 2021, there were more than 260 million confirmed cases, including 5,224,519 deaths worldwide due to COVID-19 (https://covid19.who.int). Although the high morbidity and mortality rate of COVID-19 has accelerated the development of vaccines, the emergence of pandemic SARS-CoV-2 variants remains a serious global health problem.

SARS-CoV-2, which is closely related to SARS-CoV-1 (an earlier type of SARS-CoV occurred in 2002) with 79% genetic similarity (Yan et al., 2020), is a positive-sense single-stranded RNA virus belonging to the subgenus *Sarbecovirus* of the genus *Betacoronavirus* in the family *Coronaviridae*. Upon entry into host cells, SARS-CoV-2 produces two polyproteins that are subsequently proteolytically processed into 16 non-structural proteins (Nsp1-Nsp16) which further form viral replicase–transcriptase complex in double-membrane vesicles (V'Kovski et al., 2021). SARS-CoV-2 genome also encodes four structural proteins including spike (S), envelope (E), membrane (M) and nucleocapsid (N), and nine accessory proteins including Orf3a, 3b, 6, 7a, 7b, 8, 9b, 9c, and 10 (Chan et al., 2020). Although accessory proteins of SARS-CoV-2 are not necessary for viral invasion and replication, they play critical roles in immune evasion (Lowery et al., 2021), as seen in other coronaviruses (Kopecky-Bromberg et al., 2007). For instance, Orf3b and Orf6 from SARS-CoV-1 were found to antagonize with type-I interferon signaling during SARS-CoV-1 infection (Liu et al., 2014).

Accumulating evidence indicates that dysregulated innate immune response is assigned an important role in the pathogenesis of highly pathogenic coronaviruses such as SARS-CoV-1 and Middle East respiratory syndrome coronaviruse (MERS-CoV) (Cameron et al., 2007; Channappanavar et al., 2016; Channappanavar et al., 2019). A diminished and delayed type I IFN (IFN-I) response in patients with moderate-to-severe COVID-19 is demonstrated in recent findings (Arunachalam et al., 2020; Yu et al., 2020; Galani et al., 2021). Furthermore, SARS-CoV-2 has been proved to use diverse strategies to antagonize the IFN response by interaction mapping and *in vitro* overexpression assays (Blanco-Melo et al., 2020; Gordon et al., 2020; Lei et al., 2020; Lowery, et al., 2021). Among these, accessory protein Orf6 from SARS-CoV-2 is found to suppress IFN signaling by targeting the ribonucleic acid export 1 (Rae1)–nucleoporin 98 (Nup98) complex (Miorin et al., 2020; Addetia et al., 2021; Kato et al., 2021), but the molecular mechanism of this process remains to be fully elucidated.

Rae1 is a messenger RNA transport factor that can anchor to the Gle2-binding sequence (GLEBS) motif of Nup98 (Nup98_GLEBS_) at the nuclear pore complex (NPC) (Pritchard et al., 1999). The Rae1–Nup98 complex not only contributes to mRNA nuclear export but also plays functional roles at several stages of the cell cycle (Jeganathan et al., 2005). It has been reported that some viruses from unrelated species, such as vesicular stomatitis virus (VSV) and Kaposi’s sarcoma-associated herpesvirus (KSHV), can target the Rae1–Nup98 complex and suppress the host immune response (Quan et al., 2014; Feng et al., 2020). These different viruses encode specific proteins to directly interact with the Rae1–Nup98 complex. The crystal structures of viral proteins in complex with the Rae1–Nup98_GLEBS_ heterodimer have been reported (Quan et al., 2014; Feng et al., 2020).

Orf6 from SARS-CoV-2 has 61 residues and was detected partially colocalizing with Golgi apparatus (Zhang et al., 2020). Coronaviruses from subgenus *Sarbecovirus* encode the *ORF6* gene uniquely, and no orthologues have been found in other members from the genus *Betacoronaviruses* (Kimura et al., 2021). Gordon et al. identified the interactions between SARS-CoV-2 Orf6 and the Rae1–Nup98 complex for the first time in 2020, and some other convincing evidence was published thereafter (Gordon et al., 2020; Lei et al., 2020; Miorin et al., 2020; Addetia et al., 2021). It has been shown that SARS-CoV-2 Orf6 can prevent the nuclear export of host mRNA and further downregulate the expression of newly transcribed transcripts. Moreover, the C-terminal tail (CTT) of Orf6 is critical for its interaction with the Rae1–Nup98 complex and antagonism of IFN signaling (Lei et al., 2020; Miorin et al., 2020; Addetia et al., 2021). It has been suggested that the host-virus interactions could be applied to develop novel antiviral agents and repurpose existing drugs in recent studies (Gordon et al., 2020). Hence, knowledge of the molecular details of SARS-CoV-2 Orf6 targeting nuclear export is important to exploit antiviral small-molecule drugs (e.g., small molecules that modulate host nuclear export) treating COVID-19 (Lee et al., 2021).

In this study, we assessed the interactions between isolated SARS-CoV-2 Orf6 and the Rae1–Nup98 complex through isothermal titration calorimetry (ITC) and found that Orf6 is bound to the Rae1–Nup98 complex with a nanomolar *K*_D_. We showed that Orf6 competed for *in vitro* binding of single-stranded RNA (ssRNA) to the Rae1–Nup98 complex through electrophoretic mobility shift assay (EMSA). To better understand the molecular basis of SARS-CoV-2 Orf6 interacting with the Rae1–Nup98 complex, we determined the crystal structures of SARS-CoV-2 Orf6 and SARS-CoV-1 Orf6 in complex with the Rae1–Nup98_GLEBS_ heterodimer, respectively. In both structures, Orf6 occupies the same mRNA-binding pocket of the Rae1–Nup98 complex *via* interactions with conserved residues. Our structural data depicted the key binding motif in the CTT of Orf6 including the buried methionine residue, which was further confirmed in mutagenesis studies. Structural comparisons revealed common features for the Rae1-Nup98 complex hijacking by multiple viruses. Altogether our data provide a structural mechanistic understanding of SARS-CoVs Orf6 interacting with the Rae1–Nup98 complex to antagonize host interferon signaling by interfering with nuclear transportation of host mRNA.

## Materials and Methods

### Materials

Polyethylene glycol (PEG3350) for crystallization was purchased from Solarbio. Other reagents used in this study were obtained from Aladdin. A 21-mer peptide (residues 41–61) of SARS-CoV-2 Orf6, a 22-mer peptide (residues 42–63) of SARS-CoV-1 Orf6 and the M58A/M58R mutants of SARS-CoV-2 Orf6_CTT_ were synthesized from KS-V Peptide Co., Hefei, China. Single-stranded RNA used in this study was synthesized from GenScript.

### Plasmid Construction

The gene encoding full-length ribonucleic acid export 1 (Rae1, residues 1–368) and the Gle2-binding sequence (GLEBS) motif of Nup98 (residues 157–213) were synthesized and inserted into the pFastBacDual vector (Invitrogen) downstream of the p10 promoter region and the polyhedrin promoter region, respectively, for co-expression in *Spodoptera frugiperda* (*Sf*9) cells. Rae1 and Nup98_GLEBS_ were individually expressed with a non-cleavable C-terminal and N-terminal deca-histidine, respectively.

### Protein Expression and Purification

For co-expression of the Rae1–Nup98_GLEBS_ complex, *Sf*9 cells were infected with recombinant baculoviruses and grown in serum-free SF900 medium (Gibco) for 3 days. The cells were harvested by centrifugation (800 × g, 30 min) and resuspended in lysis buffer containing 50 mM Tris-HCl pH 8.0, 300 mM NaCl, 0.1 mM TCEP, 5% glycerol, and 1 mM phenylmethylsulfonyl fluoride (PMSF). Then the cells were lysed with a high-pressure cell disrupter (JNBIO), and the lysate was centrifuged for 60 min at 18,000 × g. The supernatant was loaded onto a HisTrap HP affinity column (GE Healthcare), eluted in 50 mM Tris (pH 8.0), 300 mM NaCl, and 250 mM imidazole. The protein was subsequently concentrated and loaded onto a Superdex 75 increase 10/300 GL column (GE Healthcare) to exchange the protein into crystallization buffer (20 mM Tris-HCl pH 8.0, 150 mM NaCl, 0.5 mM TCEP). The peptides of SARS-CoV-2 Orf6_CTT_ and SARS-CoV-1 Orf6_CTT_ were dissolved in the same crystallization buffer of the Rae1–Nup98_GLEBS_ complex. To prepare SARS-CoV-2 Orf6_CTT_–Rae1–Nup98_GLEBS_ and SARS-CoV-1 Orf6_CTT_–Rae1–Nup98_GLEBS_ ternary complexes, 1.5 equimolar amounts of two SARS-CoVs Orf6_CTT_ were mixed with the Rae1–Nup98_GLEBS_ complex and incubated for 30 min at room temperature. The complexes were finally concentrated to ∼10 mg/ml, respectively for crystallization.

### Crystallization and Structure Determination

SARS-CoVs Orf6_CTT_–Rae1–Nup98_GLEBS_ complex crystals were obtained at 20°C by vapor diffusion in sitting drops using 1 μl of the protein (10 mg/ml) and 1 μl of a reservoir solution consisting of 100 mM sodium citrate pH 5.5, 20% PEG 3350. Crystals were formed within 2 weeks and subsequently cryoprotected in the reservoir solution with the addition of 20% glycerol and flash frozen in liquid nitrogen. Diffraction data were collected at the Shanghai Synchrotron Radiation Facility (SSRF) beamlines BL17U1 (for SARS-CoV-2 Orf6_CTT_–Rae1–Nup98_GLEBS_) and BL19U1 (for SARS-CoV-1 Orf6_CTT_–Rae1–Nup98_GLEBS_), respectively. Data were processed and scaled using HKL2000 (Otwinowski and Minor, 1997) and XDS (Kabsch, 2010).

The crystal structures of SARS-CoVs Orf6_CTT_–Rae1–Nup98_GLEBS_ complex were solved by molecular replacement (Bunkoczi and Read, 2011) with PHASER (McCoy et al., 2007) using the coordinates of Rae1–Nup98 (PDB ID 3MMY) (Ren et al., 2010) as the search model. The models of SARS-CoVs Orf6_CTT_–Rae1–Nup98_GLEBS_ were built using Coot (Emsley and Cowtan, 2004) and refined using Phenix (Adams et al., 2010). Details of the data collection and refinement statistics are summarized in Supplementary Table S1. The final models were validated by MolProbity (Chen et al., 2010). All structural figures were generated with PyMOL (Version 2.3.0 Schrödinger, LLC).

### Electrophoretic Mobility Shift Assay

Two micromolars of a degenerate decameric ssRNA oligonucleotide was incubated with increasing concentrations of the Rae1–Nup98 complex in a buffer containing 20 mM Tris pH 8.0, 150 mM NaCl and 0.5 mM TCEP, at room temperature for 5 min. Samples were separated on a 5% native PAGE gel that was prepared with 45 mM Tris, pH 7.0 (titrated with glycine to allow the Rae1–Nup98 complex to enter the gel) and pre-run in the same buffer. After electrophoresis, the RNA was visualized through the use of EnVision Multilabel Reader (Perkin Elmer).

### Isothermal Titration Calorimetry

The binding of SARS-CoVs Orf6 with the Rae1–Nup98 complex was measured by isothermal titration calorimetry (ITC) using a Micro ITC-200 calorimeter (Malvern). SARS-CoVs Orf6 peptides were dissolved in the buffer composed of 20 mM Tris-HCl pH 8.0, 150 mM NaCl, 0.5 mM TCEP. The titration was performed at 25°C by injecting 50 μl of Orf6 peptides (500 μM) into the calorimetric cell (∼300 μl) containing the Rae1–Nup98 complex at a concentration of 50 μM. The experiments involved 20 injections of SARS-CoVs Orf6 peptides into the Rae1–Nup98 sample. The heat released during the injection was obtained from the integration of the calorimetric signal. The enthalpy change (ΔH) and association constant (Ka = 1/Kd) were obtained by nonlinear regression of the data. Microcal Origin software was used for nonlinear curve fitting to a single binding site model. ITC titration was repeated at least twice for each experiment.

### Sequence Alignment, Conservation and Mutagenesis Analysis

Orf6 sequences from SARS-CoV-2 and other coronaviruses were manually downloaded from UniProt and screened for availability. Accordingly, a total of 155 SARS-CoV-2 Orf6 sequences and 98 other coronavirus Orf6 sequences were included and aligned in Jalview using MAFFT (Katoh and Standley, 2013). Frequencies of amino acids of different Orf6 sequences were analyzed using WebLogo 2.8.2 (http://weblogo.berkeley.edu). The analysis of mutations was carried out using GISAID CoVSurver (https://www.gisaid.org/epiflu-applications/covsurver-mutations-app/) with the reference sequence hCoV-19/Wuhan/WIV04/2019.

## Results

### Both SARS-CoV-2 Orf6 and SARS-CoV-1 Orf6 Can Directly Interact With the Rae1–Nup98 Complex to Disrupt Its RNA-Binding Capacity

**FIGURE 1:**
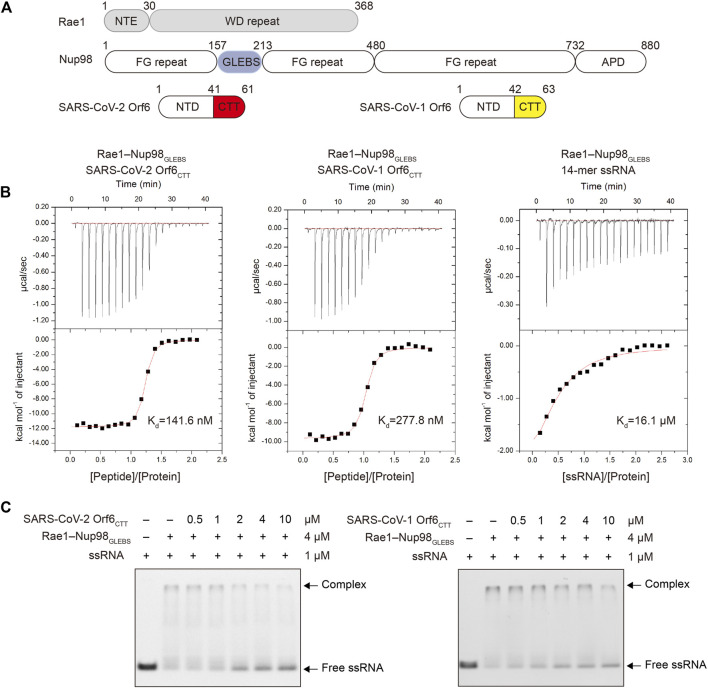
SARS-CoV-2 Orf6 and SARS-CoV-1 Orf6 bind to the Rae1–Nup98 complex with high affinity. **(A)** Domain organization of Rae1, Nup98, SARS-CoV-2 Orf6 and SARS-CoV-1 Orf6. **(B)** Binding isotherms for the interactions of SARS-CoV-2 Orf6_CTT_ **(left)**, SARS-CoV-1 Orf6_CTT_ **(middle)** and 14-mer poly(U) ssRNA **(right)** with the Rae1–Nup98 complex. **(C)** Competitive EMSA assays with SARS-CoVs Orf6, ssRNA and the Rae1–Nup98 complex. After preincubation of FAM-labeled 14-mer poly(U) ssRNA and the Rae1–Nup98 complex, the mixture was further incubated with increasing amounts of SARS-CoV-2 **(left)** and SARS-CoV-1** (right)** Orf6 peptides and analyzed by EMSA assays.

It has been found that Orf6 from SARS-CoV-2 and SARS-CoV-1 can antagonize IFN-I and the inflammatory response (Kopecky-Bromberg et al., 2007; Lei et al., 2020). Previous colocalization, coimmunoprecipitation (co-IP) and pull-down experiments have shown that SARS-CoV-2 Orf6 can directly interact with the Rae1–Nup98 complex through its C-terminal tail (Miorin et al., 2020). Given that Orf6 from SARS-CoV-1 has high sequence similarity with SARS-CoV-2 (∼85.7%), we anticipated that Orf6 from both viruses could interact with the Rae1–Nup98 complex. To confirm this interaction, we synthesized the 21-mer peptide of Orf6_CTT_ for SARS-CoV-2 and the 22-mer peptide of Orf6_CTT_ for SARS-CoV-1, and recombinantly expressed and purified the Rae1–Nup98_GLEBS_ complex by using the baculovirus–insect cell system (Figure 1A, Supplementary Figure S1). To assess the binding affinities of Orf6 to the Rae1–Nup98 complex, we performed ITC analysis. The results showed that binding of each SARS-CoV Orf6_CTT_ to the Rae1–Nup98_GLEBS_ complex occurred at a 1:1 ratio in the nanomolar range (*K*_d_ = 277.8 nM for SARS-CoV-1 Orf6 and *K*_d_ = 141.6 nM for SARS-CoV-2 Orf6), which is approximately 50- to 100-fold higher than that for a 14-mer poly(U) ssRNA binding to Rae1–Nup98_GLEBS_ (Figure 1B). ITC results quantitively described that SARS-CoV-2 Orf6_CTT_ interacts with the Rae1–Nup98_GLEBS_ complex with higher affinities compared to SARS-CoV-1 Orf6_CTT_, which is consistent with the results of the GFP pull-down assay described previously (Addetia et al., 2021).

Next, we tested whether SARS-CoV-1 and SARS-CoV-2 Orf6_CTT_ can inhibit mRNA export through binding to the Rae1–Nup98_GLEBS_ complex using EMSA. The results showed that both SARS-CoV-2 Orf6_CTT_ and SARS-CoV-1 Orf6_CTT_ competed with ssRNA for binding to the Rae1–Nup98_GLEBS_ in a concentration-dependent manner (Figure 1C). Collectively, these results demonstrate that the Orf6_CTT_ from SARS-CoV-2 and SARS-CoV-1 can closely contact the Rae1–Nup98_GLEBS_ complex and further inhibit RNA binding.

### SARS-CoV-2 Orf6 and SARS-CoV-1 Orf6 Share the Same Binding Site on Rae1

To better understand the molecular basis of SARS-CoVs Orf6 inhibiting mRNA nuclear export, we determined the crystal structures of SARS-CoV-2 Orf6_CTT_–Rae1–Nup98_GLEBS_ and SARS-CoV-1 Orf6_CTT_–Rae1–Nup98_GLEBS_ to resolutions of 2.8 and 2.5 Å, respectively. Both structures were solved by molecular replacement in the space group *C*2, with four Orf6_CTT_–Rae1–Nup98_GLEBS_ heterotrimers in one asymmetric unit bearing virtually identical confirmations. The final structures were reliably refined with good stereochemical parameters (*R*_work_ and *R*_free_ values of 0.21 and 0.25 for SARS-CoV-2 Orf6_CTT_–Rae1–Nup98_GLEBS_, while 0.19 and 0.23 for SARS-CoV-1 Orf6_CTT_–Rae1–Nup98_GLEBS_). For detailed crystallographic statistics, see Supplementary Table S1.

**FIGURE 2:**
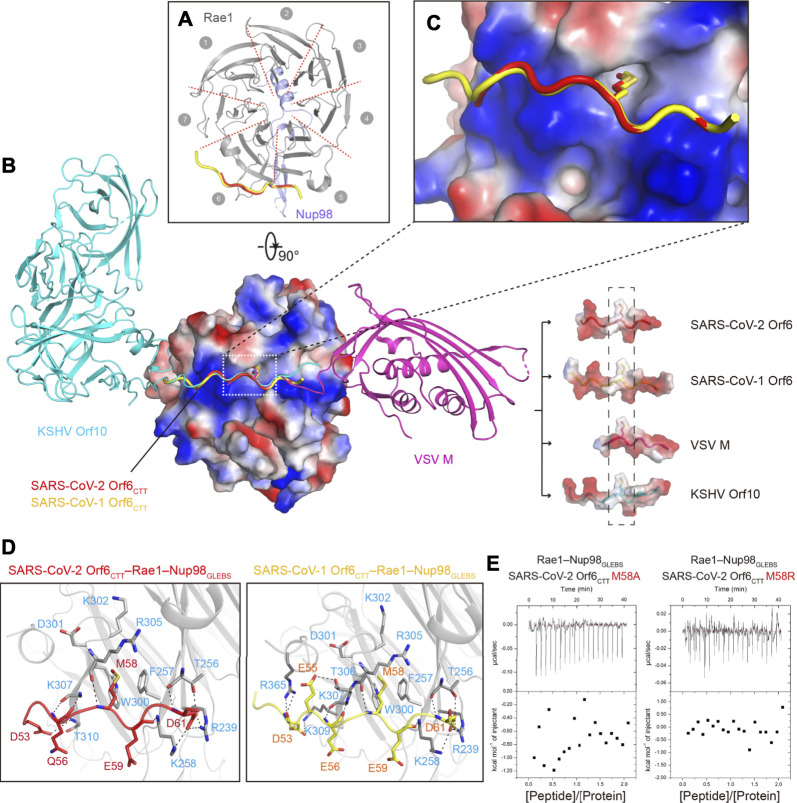
Crystal structures of SARS-CoVs Orf6_CTT_–Rae1–Nup98_GLEBS_. **(A)** Ribbon representation of SARS-CoVs Orf6_CTT_–Rae1–Nup98_GLEBS_ in top view, with the same color code as in Figure 1A. Structures of SARS-CoV-2 Orf6_CTT_ and SARS-CoV-1 Orf6_CTT_ are overlaid to show the same binding site on the Rae1–Nup98 complex. **(B)** Structural superposition of SARS-CoVs Orf6_CTT_, VSV M–Rae1–Nup98_GLEBS_ (PDBID: 4OWR), and KSHV Orf10–Rae1–Nup98_GLEBS_ (PDBID: 7BYF). VSV M and KSHV Orf10 are colored with magentas and cyan, respectively. The Rae1–Nup98_GLEBS_ complex and the interacting region of four viral proteins are shown in electrostatic potential surface representation. The conserved methionine residues are shown as sticks. **(C)** Surface electrostatic potential plot of the Rae1–Nup98 complex to show the environment around M58 of SARS-CoVs Orf6. **(D)** Residues of Rae1 participating in interactions with SARS-CoV-2 Orf6 **(left)** and SARS-CoV-1 **(right)** are shown as stick models in gray. Hydrogen bonds are shown as black dashed lines. **(E)** Binding isotherms for the interaction of SARS-CoV-2 Orf6 mutants (M58A and M58R) with the Rae1–Nup98_GLEBS_ complex.

There are no significant conformational changes for the Rae1–Nup98_GLEBS_ moiety in structures of either Orf6_CTT_–Rae1–Nup98_GLEBS_ heterotrimers or the Rae1–Nup98_GLEBS_ heterodimer reported previously (PDBID:3MMY) (Ren et al., 2010). The composite omit map of two structures unequivocally showed that residues 53–61 of SARS-CoV-2 Orf6 and residues 50–62 of SARS-CoV-1 Orf6 accommodate the same site of Rae1 (Supplementary Figure S2). Each peptide of Orf6_CTT_ adopts an identical elongated loop conformation (0.43 Å RMSD for 9 Cαs) and binds to the Rae1–Nup98_GLEBS_ heterodimer alongside blades 5 to 6 of Rae1 β-propeller (Figure 2A). The binding site has a highly positive electrostatic potential which was proposed to be potential for RNA-binding (Figures 2B,C) (Ren et al., 2010). The electron densities for other residues in peptides of Orf6_CTT_ were poorly defined that no atoms could be positioned, suggesting a highly flexible region of the peptide without any close contact to Rae1 or Nup98.

### Interactions Between SARS-CoVs Orf6_CTT_ and The Rae1–Nup98_GLEBS_ Complex

The detailed interactions between SARS-CoVs Orf6_CTT_ and the Rae1–Nup98_GLEBS_ complex were schematized in Figure 2D. Orf6_CTT_ binds to Rae1 in the positively charged surface patch *via* key interactions that are primarily composed of hydrophobic interactions and hydrogen bonding. The sidechain of a conserved methionine (M58) in both Orf6_CTT_ inserts into the hydrophobic pocket made up of residues F257, W300, D301, K302 and R305 in Rae1, which provides high steric complementarity and buries a large surface area (Figure 2C, Supplementary Figure S3). A cluster of negatively charged or polar residues on either side of M58 forms additional hydrogen bonds to Rae1. The binding patterns of Orf6_CTT_ to the Rae1–Nup98 complex in the two SARS-CoVs remain the same due to high sequence identity, especially for the interactions mediated by Orf6_CTT_ residues M58, E59 and D61 (Figure 2D).

### The M58 of SARS-CoVs Orf6_CTT_ is Critical for Rae1 Binding

Several virus-encoded proteins were reported to directly interact with the Rae1-Nup98 complex to inhibit the export of mRNA during viral infections. Here we superimposed four structures of Rae1-Nup98_GLEBS_ targeted by the two SARS-CoV Orf6_CTT_ we solved together with VSV M and KSHV Orf10 (PDBIDs: 4OWR and 7BYF, respectively). Structural alignment analysis demonstrated that the N-terminal tail (NTT) of VSV M and the CTT of KSHV Orf10 share a similar loop conformation with peptides of Orf6_CTT_ and occupy the same binding site on Rae1–Nup98_GLEBS_ (Quan et al., 2014; Feng et al., 2020) (Figure 2B). Some common features can be summarized through alignment of SARS-CoVs Orf6_CTT_ with the NTT of VSV M and the CTT of Orf10, including the conserved methionine residue with the neighboring acidic residues (Supplementary Figure S3). The surface electrostatic potential calculation revealed that these four viral proteins possess a highly conserved methionine residue with the neighboring negatively charged residues that directly contact with the overall positive electrostatic potential patch on Rae1 (Figure 2B). The side chains of the conserved methionine residues from all four viral proteins were almost completely surrounded, where the buried surface area of methionine is ∼150 Å^2^ (Figure 2B, Supplementary Figure S3). To evaluate the contribution of Orf6_CTT_ M58 to Rae1 binding, single point mutations of SARS-CoV-2 Orf6_CTT_ M58A and M58R were generated and examined in the binding assay (Miorin et al., 2020). ITC results showed that the Orf6_CTT_ M58A/M58R mutations led to complete loss of Rae1 binding (Figure 2E), indicating that M58 of SARS-CoVs Orf6_CTT_ is critical for high-affinity Rae1 binding. In conclusion, these results demonstrate that the Rae1–Nup98 complex is a crucial target for different viruses and the residue methionine is critical for the direct tight binding to the Rae1–Nup98 complex.

### Orf6 Shows High Global Conservation Among Sarbecoviruses, Especially the C-Terminal Tail Motif

**FIGURE 3:**
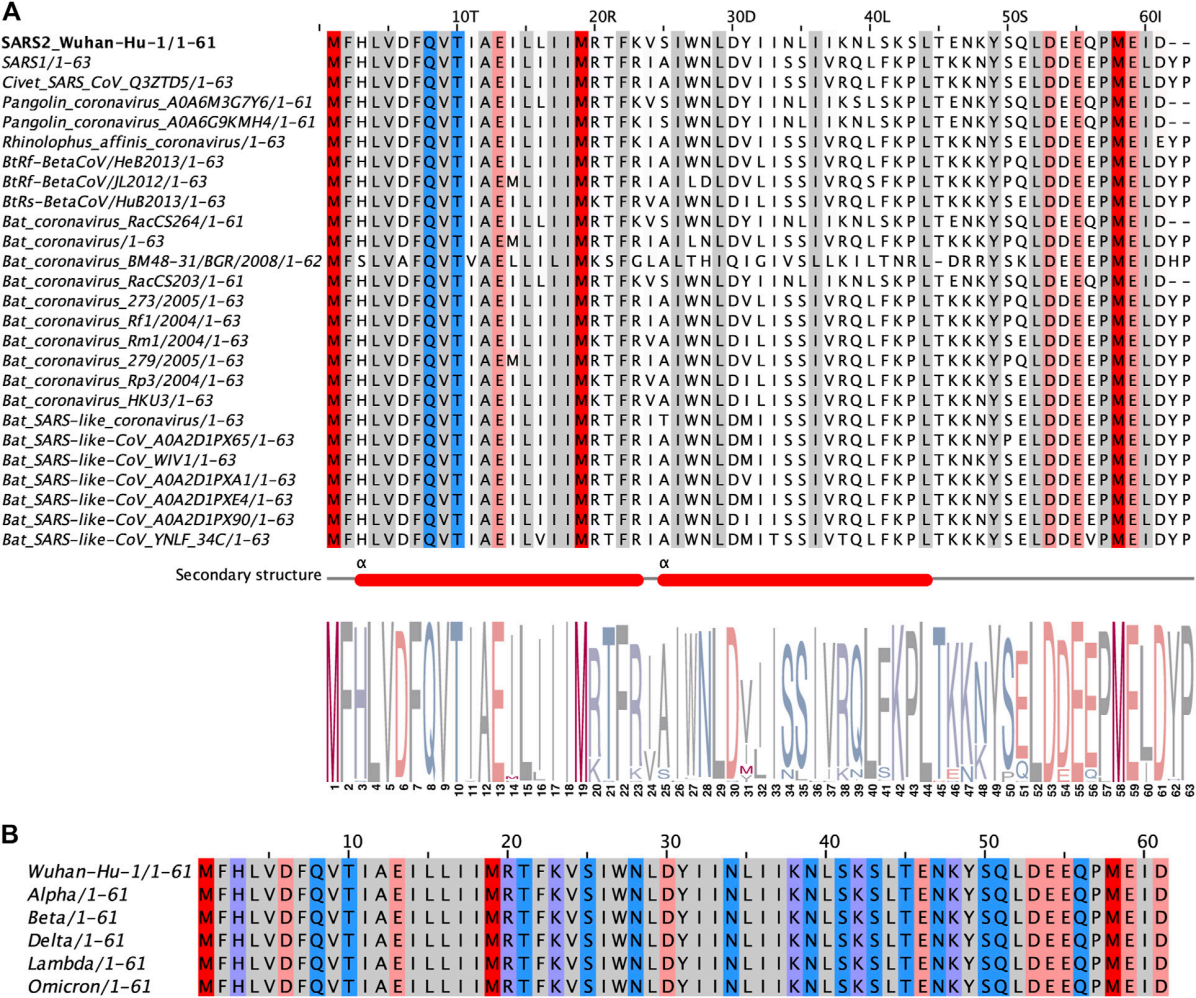
Sequence alignments of sarbecoviruses Orf6 proteins. **(A)** Alignment of representative sequences across different sarbecoviruses species. The N-terminal motif and C-terminal tail of Orf6 are highly conserved, as illustrated here by a WebLogo (http://weblogo.berkeley.edu). **(B)** Alignment of sequences from epidemic SARS-CoV-2 variants including Alpha (B.1.1.7 lineage), Beta (B.1.351 lineage), Delta (B.1.617.2 lineage), Lambda (C.37 lineage) and Omicron (B.1.1.529 lineage). Representative sequences of Orf6 homologs are aligned with respect to isolate Wuhan-Hu-1. The high sequence conservation (>95%) at each position is highlighted in different colors (Red for methionines, pink for negatively charged amino acids, purple for positively charged amino acids, blue for polar amino acids and gray for hydrophobic amino acids).

Since SARS-CoV-2 remains widespread at an alarming rate, the virus accumulated mutations in the process. To further understand which regions of Orf6 are functionally significant, we performed a sequence alignment analysis of Orf6 across sarbecoviruses from different species including civet, pangolin and bat. Orf6 shows high conservation, with the N-terminal motif and C-terminal tail of the protein (Figure 3A). Importantly, the most conserved residues in the C-terminal tail of Orf6 include D53, E55, M58, E59 and D61, which is consistent with our structural and biochemical results, suggesting that this region plays functional roles in virus pathogenicity and virulence. Besides, 155 Orf6 sequences from different SARS-CoV-2 genomes were fetched from the UniProt database, of which a total of 124 variants were observed (Supplementary Figure S4). The distribution of Orf6 variants have been summarized (Supplementary Table S2 and Supplementary Figure S5). Orf6 exhibited low variability across 155 sequences (the mutation rates of each amino acid were less than 6%) and was invariant in the recent pandemic variants including Alpha, Beta, Delta, Lambda and Omicron (Figure 3B), which implies that Orf6 may play significant roles in the replication, pathogenesis, and regulation of coronavirus.

## Discussion

The accessory protein Orf6 uniquely exists in sarbecoviruses and its function is ambiguous. SARS-CoV-1 Orf6 has been reported to interfere with interferon signaling by preventing nucleocytoplasmic transport (Frieman et al., 2007). After the outbreak of COVID-19 caused by SARS-CoV-2, Gordon and colleagues revealed a convincing interplay between SARS-CoV-2 Orf6 and the host Rae1–Nup98 complex which is responsible for nucleocytoplasmic shuttling of mRNA (Gordon et al., 2020). Several groups have also provided evidence to describe this interaction (Lei et al., 2020; Miorin et al., 2020; Addetia et al., 2021; Kato et al., 2021). Here, our crystallographic data on SARS-CoVs Orf6_CTT_–Rae1–Nup98_GLEBS_ heterotrimer directly confirmed how Orf6 from both SARS-CoV-1 and SARS-CoV-2 interacts with the Rae1–Nup98 complex (Figures 2A–C). The CTT of SARS-CoVs Orf6 represents a favorable charge complementarity to the mRNA binding groove of the Rae1–Nup98 complex, which is consistent with the high affinities of SARS-CoVs Orf6 to the Rae1–Nup98 complex (Figure 1B). Notably, the methionine (M58) of SARS-CoVs Orf6 packs into a deep hydrophobic pocket in Rae1 (Figure 2C). Further mutagenesis analyses identified this conserved methionine as a critical determinant for the binding affinity of SARS-CoV-2 Orf6 to the Rae1–Nup98 complex (Figure 2E). Additional biochemical studies showed that the binding of SARS-CoVs Orf6 to the Rae1–Nup98 complex gives rise to the displacement of ssRNA (Figure 1C). Finally, by analyzing sequences of SARS-CoV-related viruses isolated from different species, we found that the C-terminal region of Orf6 shows high global conservation, indicating its potentially vital roles in virus pathogenesis (Figure 3A). Our data support the previously established role of SARS-CoV-2 Orf6 in antagonizing mRNA nuclear export by interacting with the Rae1-Nup98 complex, and provide a structural basis to elucidate sarbecovirus Orf6 functions.

The nuclear transport of host mRNA encoding antiviral proteins is essential for innate immune signal transduction and inhibition of viral replication. Accordingly, several viruses have developed multiple strategies to counteract host mRNA export machinery. For instance, the non-structural protein 1 (NS1) of influenza A virus has been reported to form an inhibition complex with key mRNA export factors and downregulate Nup98, thus contributing to the suppression of mRNA export (Satterly et al., 2007). In addition, two well-studied examples are the M protein of VSV and Orf10 of KSHV which were found to target the Rae1–Nup98 complex and prevent mRNA nuclear export. Our structure confirms that coronaviruses also adopt the strategy of impairing host mRNA export pathways to suppress the immune response. Notably, structural data show that SARS-CoVs Orf6, VSV M and KSHV Orf10 attach to the same positive-charged groove which is considered to be the binding site of mRNA to the Rae1–Nup98 complex. Our findings highlight the common strategy by which different viruses have evolved to block interferon signaling and provide new insights into the investigation of therapeutic antiviral targets.

Recently various publications have demonstrated that SARS-CoV-2 applies a multipronged strategy to hijack the host innate immune system. For example, SARS-CoV-2 Nsp1 was found to block mRNA translation through interacting with the 18S ribosomal RNA in the mRNA entrance channel (Schubert et al., 2020). SARS-CoV-2 Nsp16 was shown to disrupt global mRNA splicing by binding to the mRNA recognition motifs of U1/U2 small nuclear RNA (Banerjee et al., 2020). Coupled with our structural and biochemical results, we support the previous observations for SARS-CoV-2 Orf6 binding to the Rae1–Nup98 complex and inhibiting immune responses. However, a recent study using an ectopic expression assay showed that SARS-CoV-2 Orf6 binds to Nup98 and has an influence on the nuclear import of STAT by disrupting karyopherin alpha 1 (KPNA1)-karyopherin beta 1 (KPNB1) docking at the NPC. In our structure, no interactions between the CTT of SARS-CoV-2 Orf6 and the GLEBS motif of Nup98 were observed, which implied that Orf6 may interact with Nup98 *via* regions other than GLEBS, possibly through residues on the CTT or NTT of Orf6. The underlying mechanism remains to be fully investigated.

In summary, our results provide detailed structural and molecular mechanisms of both SARS-CoV-2 and SARS-CoV-1 Orf6 targeting the Rae1–Nup98 complex, which may subsequently mediate the inhibition of mRNA nuclear export and ultimately antagonize host interferon signaling.

## Data Availability

The atomic coordinates have been deposited in the Protein Data Bank under the codes ID 7VPH (SARS-CoV-2 Orf6_CTT_–Rae1–Nup98_GLEBS_) and 7VPG (SARS-CoV-1 Orf6_CTT_–Rae1–Nup98_GLEBS_).
