# Supplementary material for: Molecular Mechanism of SARS-CoVs Orf6 Targeting the Rae1–Nup98 Complex to Compete With mRNA Nuclear Export
Source: Front Mol Biosci. 2022 Jan 12;8:813248. doi: 10.3389/fmolb.2021.813248 (PMC8790125; doi:10.3389/fmolb.2021.813248)
Supplement: Supplementary file 1 [file DataSheet2.PDF]

Preliminary Full wwPDB X-ray Structure Validation Report (i)

Dec 8, 2021 – 09:56 PM JST

Deposition ID : D_1300025134

This is a Preliminary Full wwPDB X-ray Structure Validation Report.

This report is produced by the wwPDB Deposition System during initial deposition but before annotation of the structure.

We welcome your comments at *validation@mail.wwpdb.org*
A user guide is available at
https://www.wwpdb.org/validation/2017/XrayValidationReportHelp
with specific help available everywhere you see the (i) symbol.

PRELIMINARY VALIDATION REPORT

The following versions of software and data (see references (i)) were used in the production of this report:

MolProbity	:	4.02b-467
Xtriage (Phenix)	:	1.13
EDS	:	2.24
Percentile statistics	:	20191225.v01 (using entries in the PDB archive December 25th 2019)
Refmac	:	5.8.0158
CCP4	:	7.0.044 (Gargrove)
Ideal geometry (proteins)	:	Engh & Huber (2001)
Ideal geometry (DNA, RNA)	:	Parkinson et al. (1996)
Validation Pipeline (wwPDB-VP)	:	2.24

1 Overall quality at a glance (i)

The following experimental techniques were used to determine the structure:
X-RAY DIFFRACTION

The reported resolution of this entry is 2.80 Å.

Percentile scores (ranging between 0-100) for global validation metrics of the entry are shown in the following graphic. The table shows the number of entries on which the scores are based.

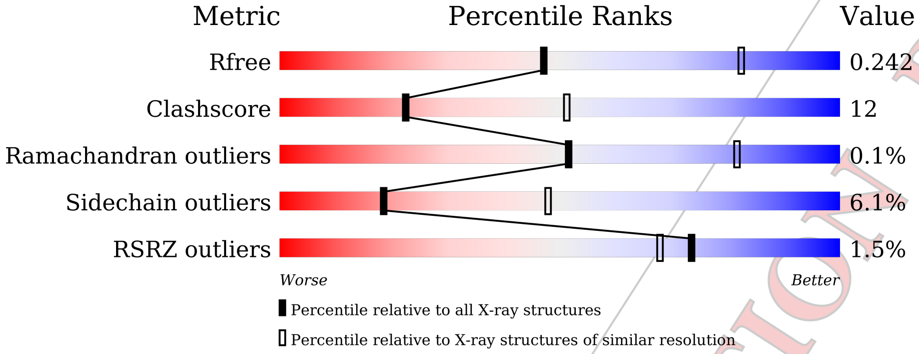

Metric	Whole archive (#Entries)	Similar resolution (#Entries, resolution range(Å))
R_{free}	130704	3140 (2.80-2.80)
Clashscore	141614	3569 (2.80-2.80)
Ramachandran outliers	138981	3498 (2.80-2.80)
Sidechain outliers	138945	3500 (2.80-2.80)
RSRZ outliers	127900	3078 (2.80-2.80)

The table below summarises the geometric issues observed across the polymeric chains and their fit to the electron density. The red, orange, yellow and green segments of the lower bar indicate the fraction of residues that contain outliers for >=3, 2, 1 and 0 types of geometric quality criteria respectively. A grey segment represents the fraction of residues that are not modelled. The numeric value for each fraction is indicated below the corresponding segment, with a dot representing fractions <=5% The upper red bar (where present) indicates the fraction of residues that have poor fit to the electron density. The numeric value is given above the bar.

Mol	Chain	Length	Quality of chain
1	A	339	72% 27% •
2	B	51	4% 80% 18% •
3	C	337	% 71% 24% • •
4	D	44	75% 25%
5	E	336	73% 25% •
6	F	54	83% 17%

Continued on next page...

PRELIMINARY VALIDATION REPORT

Continued from previous page...

Mol	Chain	Length	Quality of chain
7	G	336	2% / 66% 30% •
8	H	46	11% / 61% 39%
9	I	9	78% 11% 11%
9	K	9	33% / 67% 33%
9	X	9	33% / 78% 22%
10	J	8	88% 12%

PRELIMINARY VALIDATION REPORT

2 Entry composition (i)

There are 11 unique types of molecules in this entry. The entry contains 12571 atoms, of which 0 are hydrogens and 0 are deuteriums.

In the tables below, the ZeroOcc column contains the number of atoms modelled with zero occupancy, the AltConf column contains the number of residues with at least one atom in alternate conformation and the Trace column contains the number of residues modelled with at most 2 atoms.

- Molecule 1 is a protein called mRNA export factor.

Mol	Chain	Residues	Atoms					ZeroOcc	AltConf	Trace
			Total	C	N	O	S			
1	A	339	2681	1693	470	500	18	0	0	0

There are 10 discrepancies between the modelled and reference sequences:

Chain	Residue	Modelled	Actual	Comment	Reference
A	369	HIS	-	expression tag	UNP P78406
A	370	HIS	-	expression tag	UNP P78406
A	371	HIS	-	expression tag	UNP P78406
A	372	HIS	-	expression tag	UNP P78406
A	373	HIS	-	expression tag	UNP P78406
A	374	HIS	-	expression tag	UNP P78406
A	375	HIS	-	expression tag	UNP P78406
A	376	HIS	-	expression tag	UNP P78406
A	377	HIS	-	expression tag	UNP P78406
A	378	HIS	-	expression tag	UNP P78406

- Molecule 2 is a protein called Isoform 3 of Nuclear pore complex protein Nup98-Nup96.

Mol	Chain	Residues	Atoms					ZeroOcc	AltConf	Trace
			Total	C	N	O	S			
2	B	51	405	247	69	86	3	0	0	0

There are 11 discrepancies between the modelled and reference sequences:

Chain	Residue	Modelled	Actual	Comment	Reference
B	147	MET	-	initiating methionine	UNP P52948
B	148	HIS	-	expression tag	UNP P52948
B	149	HIS	-	expression tag	UNP P52948
B	150	HIS	-	expression tag	UNP P52948
B	151	HIS	-	expression tag	UNP P52948
B	152	HIS	-	expression tag	UNP P52948

Continued on next page...

PRELIMINARY VALIDATION REPORT

Continued from previous page...

Chain	Residue	Modelled	Actual	Comment	Reference
B	153	HIS	-	expression tag	UNP P52948
B	154	HIS	-	expression tag	UNP P52948
B	155	HIS	-	expression tag	UNP P52948
B	156	HIS	-	expression tag	UNP P52948
B	157	HIS	-	expression tag	UNP P52948

- Molecule 3 is a protein called mRNA export factor.

Mol	Chain	Residues	Atoms					ZeroOcc	AltConf	Trace
			Total	C	N	O	S			
3	C	337	2674	1690	470	496	18	0	1	0

There are 10 discrepancies between the modelled and reference sequences:

Chain	Residue	Modelled	Actual	Comment	Reference
C	369	HIS	-	expression tag	UNP P78406
C	370	HIS	-	expression tag	UNP P78406
C	371	HIS	-	expression tag	UNP P78406
C	372	HIS	-	expression tag	UNP P78406
C	373	HIS	-	expression tag	UNP P78406
C	374	HIS	-	expression tag	UNP P78406
C	375	HIS	-	expression tag	UNP P78406
C	376	HIS	-	expression tag	UNP P78406
C	377	HIS	-	expression tag	UNP P78406
C	378	HIS	-	expression tag	UNP P78406

- Molecule 4 is a protein called Isoform 3 of Nuclear pore complex protein Nup98-Nup96.

Mol	Chain	Residues	Atoms					ZeroOcc	AltConf	Trace
			Total	C	N	O	S			
4	D	44	353	217	61	73	2	0	0	0

There are 11 discrepancies between the modelled and reference sequences:

Chain	Residue	Modelled	Actual	Comment	Reference
D	147	MET	-	initiating methionine	UNP P52948
D	148	HIS	-	expression tag	UNP P52948
D	149	HIS	-	expression tag	UNP P52948
D	150	HIS	-	expression tag	UNP P52948
D	151	HIS	-	expression tag	UNP P52948
D	152	HIS	-	expression tag	UNP P52948
D	153	HIS	-	expression tag	UNP P52948

Continued on next page...

PRELIMINARY VALIDATION REPORT

Continued from previous page...

Chain	Residue	Modelled	Actual	Comment	Reference
D	154	HIS	-	expression tag	UNP P52948
D	155	HIS	-	expression tag	UNP P52948
D	156	HIS	-	expression tag	UNP P52948
D	157	HIS	-	expression tag	UNP P52948

- Molecule 5 is a protein called mRNA export factor.

Mol	Chain	Residues	Atoms					ZeroOcc	AltConf	Trace
			Total	C	N	O	S			
5	E	336	2658	1681	465	494	18	0	0	0

There are 10 discrepancies between the modelled and reference sequences:

Chain	Residue	Modelled	Actual	Comment	Reference
E	369	HIS	-	expression tag	UNP P78406
E	370	HIS	-	expression tag	UNP P78406
E	371	HIS	-	expression tag	UNP P78406
E	372	HIS	-	expression tag	UNP P78406
E	373	HIS	-	expression tag	UNP P78406
E	374	HIS	-	expression tag	UNP P78406
E	375	HIS	-	expression tag	UNP P78406
E	376	HIS	-	expression tag	UNP P78406
E	377	HIS	-	expression tag	UNP P78406
E	378	HIS	-	expression tag	UNP P78406

- Molecule 6 is a protein called Isoform 3 of Nuclear pore complex protein Nup98-Nup96.

Mol	Chain	Residues	Atoms					ZeroOcc	AltConf	Trace
			Total	C	N	O	S			
6	F	54	423	259	72	89	3	0	0	0

There are 11 discrepancies between the modelled and reference sequences:

Chain	Residue	Modelled	Actual	Comment	Reference
F	147	MET	-	initiating methionine	UNP P52948
F	148	HIS	-	expression tag	UNP P52948
F	149	HIS	-	expression tag	UNP P52948
F	150	HIS	-	expression tag	UNP P52948
F	151	HIS	-	expression tag	UNP P52948
F	152	HIS	-	expression tag	UNP P52948
F	153	HIS	-	expression tag	UNP P52948
F	154	HIS	-	expression tag	UNP P52948

Continued on next page...

PRELIMINARY VALIDATION REPORT

Continued from previous page...

Chain	Residue	Modelled	Actual	Comment	Reference
F	155	HIS	-	expression tag	UNP P52948
F	156	HIS	-	expression tag	UNP P52948
F	157	HIS	-	expression tag	UNP P52948

- Molecule 7 is a protein called mRNA export factor.

Mol	Chain	Residues	Atoms					ZeroOcc	AltConf	Trace
7	G	336	Total 2660	C 1683	N 463	O 496	S 18	4	1	0

There are 10 discrepancies between the modelled and reference sequences:

Chain	Residue	Modelled	Actual	Comment	Reference
G	369	HIS	-	expression tag	UNP P78406
G	370	HIS	-	expression tag	UNP P78406
G	371	HIS	-	expression tag	UNP P78406
G	372	HIS	-	expression tag	UNP P78406
G	373	HIS	-	expression tag	UNP P78406
G	374	HIS	-	expression tag	UNP P78406
G	375	HIS	-	expression tag	UNP P78406
G	376	HIS	-	expression tag	UNP P78406
G	377	HIS	-	expression tag	UNP P78406
G	378	HIS	-	expression tag	UNP P78406

- Molecule 8 is a protein called Isoform 3 of Nuclear pore complex protein Nup98-Nup96.

Mol	Chain	Residues	Atoms					ZeroOcc	AltConf	Trace
8	H	46	Total 369	C 227	N 64	O 76	S 2	0	0	0

There are 11 discrepancies between the modelled and reference sequences:

Chain	Residue	Modelled	Actual	Comment	Reference
H	147	MET	-	initiating methionine	UNP P52948
H	148	HIS	-	expression tag	UNP P52948
H	149	HIS	-	expression tag	UNP P52948
H	150	HIS	-	expression tag	UNP P52948
H	151	HIS	-	expression tag	UNP P52948
H	152	HIS	-	expression tag	UNP P52948
H	153	HIS	-	expression tag	UNP P52948
H	154	HIS	-	expression tag	UNP P52948
H	155	HIS	-	expression tag	UNP P52948

Continued on next page...

PRELIMINARY VALIDATION REPORT

Continued from previous page...

Chain	Residue	Modelled	Actual	Comment	Reference
H	156	HIS	-	expression tag	UNP P52948
H	157	HIS	-	expression tag	UNP P52948

- Molecule 9 is a protein called ORF6 protein.

Mol	Chain	Residues	Atoms	ZeroOcc	AltConf	Trace
9	I	9	Total C N O S<br>75 44 10 20 1	0	0	0
9	K	9	Total C N O S<br>75 44 10 20 1	0	0	0
9	X	9	Total C N O S<br>75 44 10 20 1	0	0	0

- Molecule 10 is a protein called ORF6 protein.

Mol	Chain	Residues	Atoms	ZeroOcc	AltConf	Trace
10	J	8	Total C N O S<br>67 40 9 17 1	0	0	0

- Molecule 11 is water.

Mol	Chain	Residues	Atoms	ZeroOcc	AltConf
11	S	56	Total O<br>56 56	0	0

PRELIMINARY VALIDATION REPORT

3 Residue-property plots ⓘ

These plots are drawn for all protein, RNA, DNA and oligosaccharide chains in the entry. The first graphic for a chain summarises the proportions of the various outlier classes displayed in the second graphic. The second graphic shows the sequence view annotated by issues in geometry and electron density. Residues are color-coded according to the number of geometric quality criteria for which they contain at least one outlier: green = 0, yellow = 1, orange = 2 and red = 3 or more. A red dot above a residue indicates a poor fit to the electron density (RSRZ > 2). Stretches of 2 or more consecutive residues without any outlier are shown as a green connector. Residues present in the sample, but not in the model, are shown in grey.

- Molecule 1: mRNA export factor

Chain A:

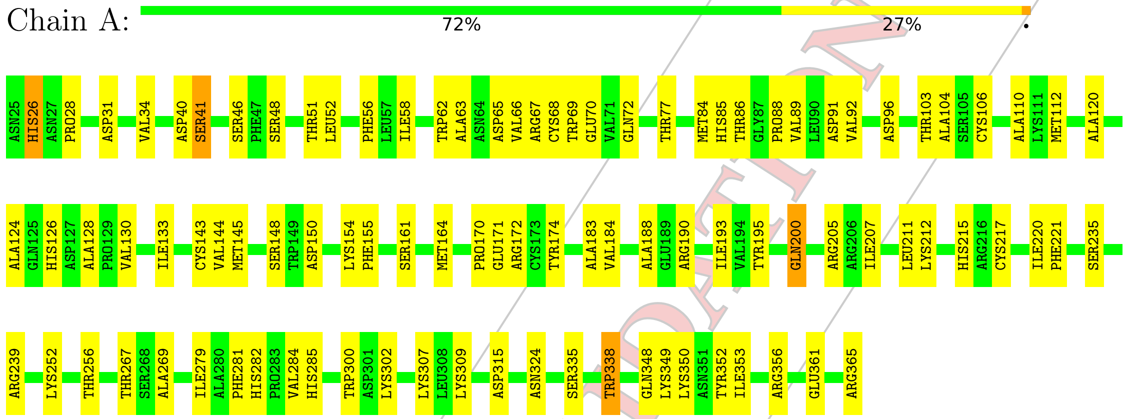

- Molecule 2: Isoform 3 of Nuclear pore complex protein Nup98-Nup96

Chain B:

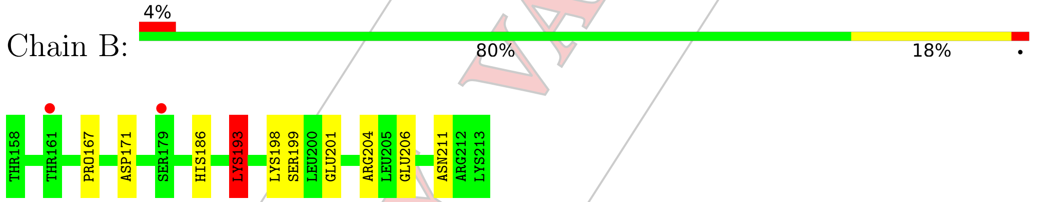

- Molecule 3: mRNA export factor

Chain C:

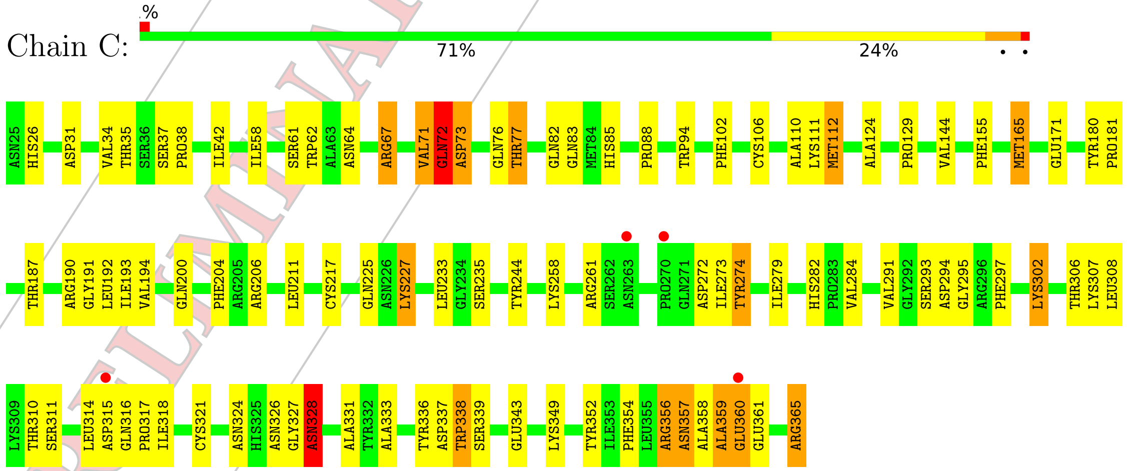

PRELIMINARY VALIDATION REPORT

• Molecule 4: Isoform 3 of Nuclear pore complex protein Nup98-Nup96

Chain D:

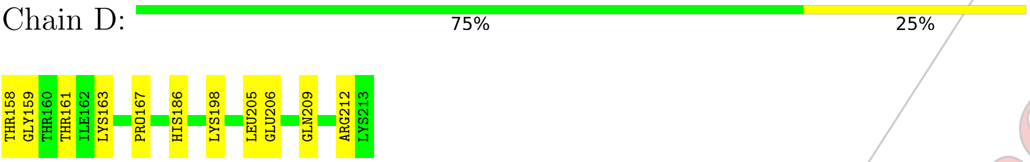

• Molecule 5: mRNA export factor

Chain E:

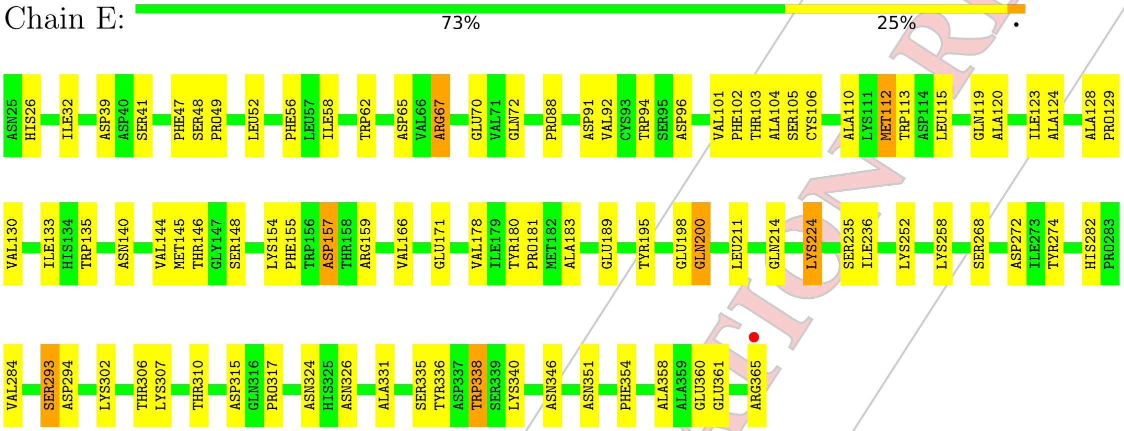

• Molecule 6: Isoform 3 of Nuclear pore complex protein Nup98-Nup96

Chain F:

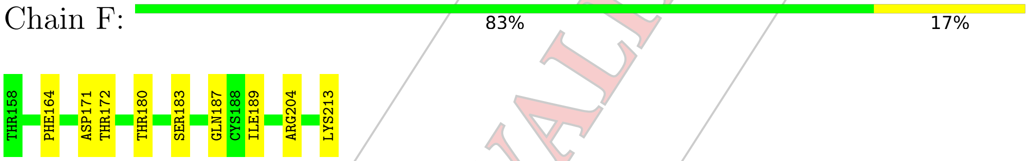

• Molecule 7: mRNA export factor

Chain G:

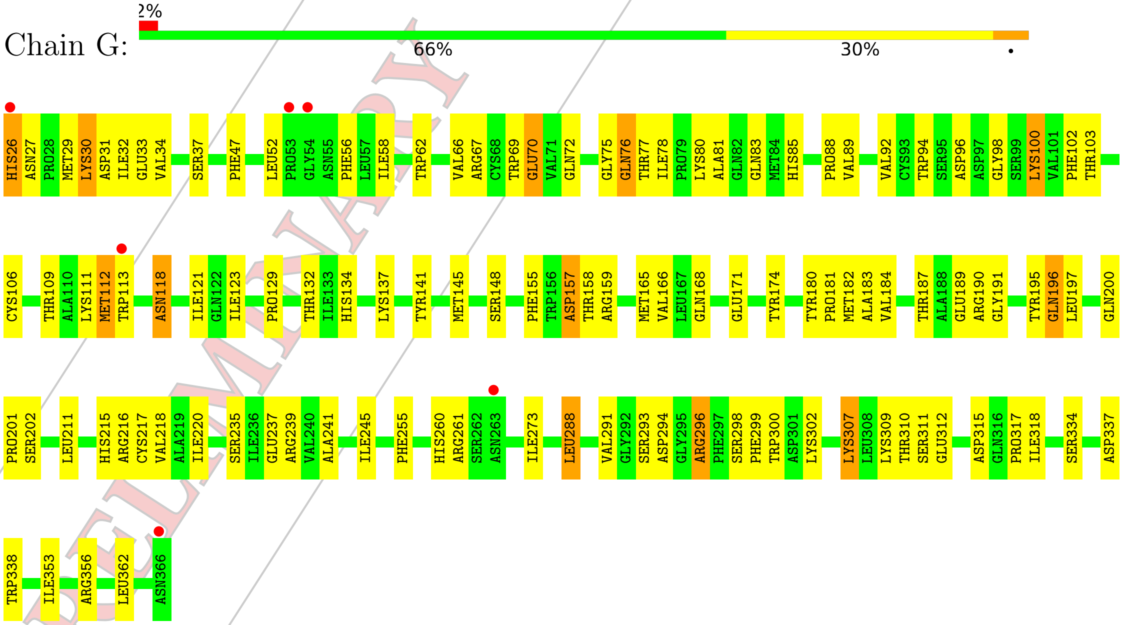

PRELIMINARY VALIDATION REPORT

• Molecule 8: Isoform 3 of Nuclear pore complex protein Nup98-Nup96

Chain H:

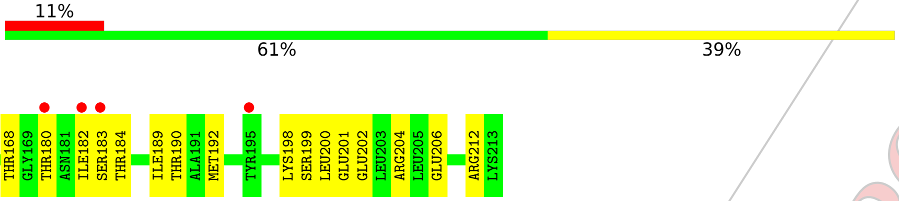

• Molecule 9: ORF6 protein

Chain I:

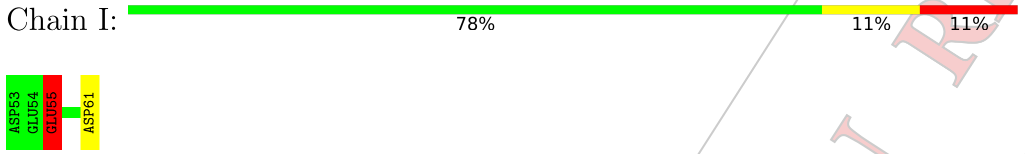

• Molecule 9: ORF6 protein

Chain K:

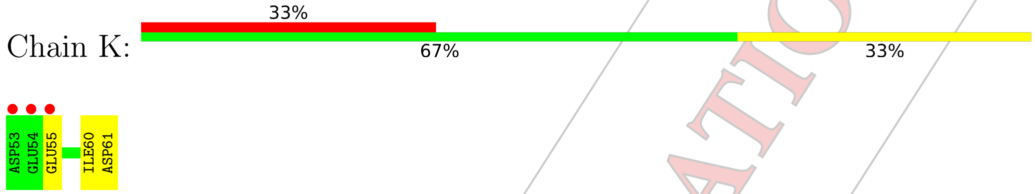

• Molecule 9: ORF6 protein

Chain X:

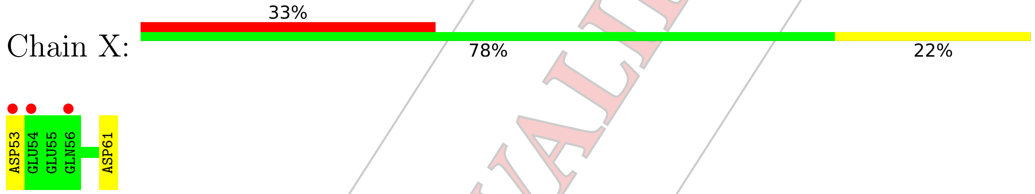

• Molecule 10: ORF6 protein

Chain J:

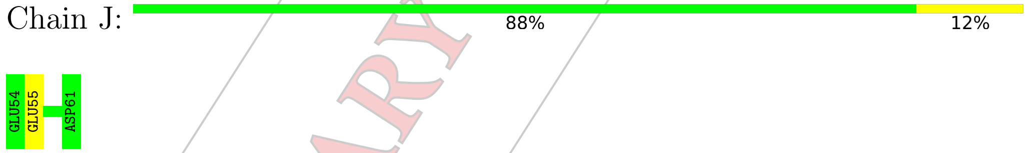

PRELIMINARY VALIDATION REPORT

4 Data and refinement statistics (i)

Property	Value	Source
Space group	C 1 2 1	Depositor
Cell constants<br>a, b, c, α, β, γ	150.66Å 103.32Å 136.59Å<br>90.00° 97.45° 90.00°	Depositor
Resolution (Å)	41.06 – 2.80<br>41.07 – 2.80	Depositor<br>EDS
% Data completeness<br>(in resolution range)	99.4 (41.06-2.80)<br>99.4 (41.07-2.80)	Depositor<br>EDS
R_{merge}	(Not available)	Depositor
R_{sym}	(Not available)	Depositor
$< I/\sigma(I) >$ [1]	1.47 (at 2.81Å)	Xtriage
Refinement program	PHENIX (1.19.2_4158: ???)	Depositor
R, R_{free}	0.192 , 0.244<br>0.191 , 0.242	Depositor<br>DCC
R_{free} test set	1992 reflections (3.89%)	wwPDB-VP
Wilson B-factor (Å^2)	58.4	Xtriage
Anisotropy	0.615	Xtriage
Bulk solvent k_{sol}(e/Å^3), B_{sol}(Å^2)	0.32 , 44.2	EDS
L-test for twinning[2]	$< \|L\| > = 0.50$, $< L^2 > = 0.33$	Xtriage
Estimated twinning fraction	No twinning to report.	Xtriage
F_o,F_c correlation	0.95	EDS
Total number of atoms	12571	wwPDB-VP
Average B, all atoms (Å^2)	73.0	wwPDB-VP

Xtriage's analysis on translational NCS is as follows: *The largest off-origin peak in the Patterson function is 3.47% of the height of the origin peak. No significant pseudotranslation is detected.*

[1] Intensities estimated from amplitudes.

[2] Theoretical values of $< |L| >$, $< L^2 >$ for acentric reflections are 0.5, 0.333 respectively for untwinned datasets, and 0.375, 0.2 for perfectly twinned datasets.

PRELIMINARY VALIDATION REPORT

5 Model quality (i)

5.1 Standard geometry (i)

The Z score for a bond length (or angle) is the number of standard deviations the observed value is removed from the expected value. A bond length (or angle) with $|Z| > 5$ is considered an outlier worth inspection. RMSZ is the root-mean-square of all Z scores of the bond lengths (or angles).

Mol	Chain	Bond lengths		Bond angles	
		RMSZ	#$\|Z\|$ >5	RMSZ	#$\|Z\|$ >5
1	A	0.56	0/2758	0.73	1/3749 (0.0%)
2	B	0.64	1/409 (0.2%)	0.71	0/547
3	C	0.57	3/2754 (0.1%)	0.89	10/3742 (0.3%)
4	D	0.50	0/357	0.72	0/476
5	E	0.51	0/2734	0.74	4/3714 (0.1%)
6	F	0.49	0/427	0.66	0/572
7	G	0.49	1/2739 (0.0%)	0.75	3/3723 (0.1%)
8	H	0.40	0/373	0.70	0/498
9	I	0.46	0/75	1.01	1/100 (1.0%)
9	K	0.40	0/75	0.66	0/100
9	X	0.53	0/75	0.81	0/100
10	J	0.45	0/67	0.69	0/89
All	All	0.53	5/12843 (0.0%)	0.77	19/17410 (0.1%)

Chiral center outliers are detected by calculating the chiral volume of a chiral center and verifying if the center is modelled as a planar moiety or with the opposite hand.A planarity outlier is detected by checking planarity of atoms in a peptide group, atoms in a mainchain group or atoms of a sidechain that are expected to be planar.

Mol	Chain	#Chirality outliers	#Planarity outliers
3	C	0	3
7	G	0	1
All	All	0	4

All (5) bond length outliers are listed below:

Mol	Chain	Res	Type	Atoms	Z	Observed(Å)	Ideal(Å)
3	C	360	GLU	CD-OE1	9.37	1.35	1.25
2	B	193	LYS	CE-NZ	6.51	1.65	1.49
3	C	328	ASN	CB-CG	6.19	1.65	1.51
3	C	72	GLN	CG-CD	5.70	1.64	1.51
7	G	196	GLN	CG-CD	5.13	1.62	1.51

PRELIMINARY VALIDATION REPORT

All (19) bond angle outliers are listed below:

Mol	Chain	Res	Type	Atoms	Z	Observed(°)	Ideal(°)
3	C	360	GLU	CG-CD-OE2	-15.36	87.58	118.30
3	C	227	LYS	CA-CB-CG	11.73	139.21	113.40
3	C	360	GLU	CG-CD-OE1	11.48	141.25	118.30
7	G	196	GLN	CA-CB-CG	10.39	136.26	113.40
3	C	227	LYS	CD-CE-NZ	9.61	133.81	111.70
1	A	349	LYS	CG-CD-CE	-7.93	88.11	111.90
3	C	72	GLN	CA-CB-CG	6.90	128.57	113.40
5	E	72	GLN	CB-CA-C	6.49	123.38	110.40
3	C	71	VAL	C-N-CA	6.31	137.47	121.70
5	E	72	GLN	CA-CB-CG	6.28	127.21	113.40
3	C	360	GLU	N-CA-C	-6.27	94.07	111.00
7	G	189	GLU	CA-CB-CG	-6.09	99.99	113.40
7	G	100	LYS	CD-CE-NZ	6.00	125.49	111.70
5	E	39	ASP	CB-CG-OD1	-5.91	112.98	118.30
9	I	55	GLU	CA-CB-CG	5.80	126.17	113.40
5	E	157	ASP	CB-CA-C	-5.58	99.23	110.40
3	C	72	GLN	CB-CA-C	5.47	121.35	110.40
3	C	328	ASN	CB-CA-C	5.40	121.19	110.40
3	C	357	ASN	N-CA-C	5.14	124.88	111.00

There are no chirality outliers.

All (4) planarity outliers are listed below:

Mol	Chain	Res	Type	Group
3	C	328	ASN	Peptide
3	C	356	ARG	Peptide
3	C	359	ALA	Peptide
7	G	26	HIS	Peptide

5.2 Too-close contacts (i)

In the following table, the Non-H and H(model) columns list the number of non-hydrogen atoms and hydrogen atoms in the chain respectively. The H(added) column lists the number of hydrogen atoms added and optimized by MolProbity. The Clashes column lists the number of clashes within the asymmetric unit, whereas Symm-Clashes lists symmetry-related clashes.

Mol	Chain	Non-H	H(model)	H(added)	Clashes	Symm-Clashes
1	A	2681	0	2575	67	0
2	B	405	0	397	9	0
3	C	2674	0	2575	62	0

Continued on next page...

PRELIMINARY VALIDATION REPORT

Continued from previous page...

Mol	Chain	Non-H	H(model)	H(added)	Clashes	Symm-Clashes
4	D	353	0	348	6	0
5	E	2658	0	2555	60	0
6	F	423	0	418	7	0
7	G	2660	0	2554	78	0
8	H	369	0	365	16	0
9	I	75	0	60	4	0
9	K	75	0	60	2	0
9	X	75	0	60	3	0
10	J	67	0	56	1	0
11	S	56	0	0	4	0
All	All	12571	0	12023	285	0

The all-atom clashscore is defined as the number of clashes found per 1000 atoms (including hydrogen atoms). The all-atom clashscore for this structure is 12.

All (285) close contacts within the same asymmetric unit are listed below, sorted by their clash magnitude.

Atom-1	Atom-2	Interatomic distance (Å)	Clash overlap (Å)
8:H:182:ILE:HG22	8:H:183:SER:H	1.33	0.93
1:A:171:GLU:HG3	1:A:172:ARG:H	1.32	0.91
3:C:328:ASN:HB2	3:C:357:ASN:OD1	1.74	0.88
3:C:34:VAL:HA	3:C:77:THR:HG21	1.56	0.86
3:C:272:ASP:HB3	3:C:274:TYR:HE2	1.40	0.85
3:C:72:GLN:OE1	3:C:73:ASP:N	2.13	0.81
1:A:171:GLU:HG2	1:A:188:ALA:HB3	1.63	0.81
7:G:293:SER:HA	7:G:317:PRO:HB3	1.64	0.80
7:G:112:MET:HE1	7:G:158:THR:HB	1.64	0.78
1:A:171:GLU:HG3	1:A:172:ARG:N	2.00	0.76
3:C:62:TRP:HA	3:C:88:PRO:HB3	1.68	0.76
7:G:165:MET:HE1	7:G:201:PRO:HD2	1.68	0.76
1:A:350:LYS:HD3	1:A:350:LYS:N	1.99	0.75
7:G:56:PHE:CE2	7:G:70:GLU:HG3	2.24	0.73
3:C:244:TYR:OH	3:C:302:LYS:NZ	2.23	0.72
1:A:267:THR:HG22	1:A:269:ALA:H	1.53	0.72
3:C:211:LEU:HD13	3:C:235:SER:HB3	1.71	0.71
3:C:272:ASP:HB3	3:C:274:TYR:CE2	2.24	0.71
3:C:307:LYS:O	9:I:55:GLU:HA	1.92	0.70
5:E:110:ALA:HB3	5:E:124:ALA:HB3	1.74	0.70
1:A:88:PRO:HD2	1:A:106:CYS:HB2	1.74	0.69
7:G:307:LYS:HE3	7:G:310:THR:OG1	1.92	0.69
5:E:88:PRO:HD2	5:E:106:CYS:HB2	1.75	0.69

Continued on next page...

PRELIMINARY VALIDATION REPORT

Continued from previous page...

Atom-1	Atom-2	Interatomic distance (Å)	Clash overlap (Å)
5:E:189:GLU:OE1	11:S:55:HOH:O	2.12	0.66
3:C:102:PHE:CE1	3:C:112:MET:HG3	2.31	0.66
3:C:357:ASN:ND2	3:C:360:GLU:OE2	2.28	0.66
7:G:34:VAL:HG13	7:G:77:THR:HG21	1.77	0.65
7:G:196:GLN:OE1	7:G:202:SER:OG	2.13	0.64
1:A:282:HIS:HD2	1:A:285:HIS:H	1.46	0.64
1:A:282:HIS:CD2	1:A:285:HIS:H	2.15	0.64
7:G:145:MET:HG3	7:G:155:PHE:CE2	2.33	0.63
5:E:145:MET:HG3	5:E:155:PHE:CE2	2.33	0.62
7:G:273:ILE:HD11	8:H:184:THR:HB	1.80	0.62
5:E:101:VAL:HG23	5:E:115:LEU:HD21	1.81	0.62
7:G:52:LEU:HD13	7:G:98:GLY:HA3	1.82	0.62
3:C:165:MET:HE1	3:C:200:GLN:HG3	1.80	0.62
7:G:337:ASP:O	8:H:200:LEU:HD23	2.00	0.62
3:C:85:HIS:CE1	3:C:111:LYS:HG3	2.34	0.62
3:C:110:ALA:HB3	3:C:124:ALA:HB3	1.82	0.62
5:E:198:GLU:O	5:E:200:GLN:N	2.33	0.62
1:A:41:SER:HG	2:B:204:ARG:HH22	1.49	0.61
7:G:29:MET:HE2	7:G:312:GLU:H	1.64	0.61
7:G:174:TYR:CZ	8:H:199:SER:HB3	2.36	0.61
1:A:143:CYS:HB3	1:A:164:MET:HE1	1.83	0.60
7:G:299:PHE:HB3	7:G:362:LEU:HD21	1.83	0.60
1:A:171:GLU:CG	1:A:188:ALA:HB3	2.30	0.60
7:G:112:MET:HE3	7:G:123:ILE:HG21	1.84	0.60
1:A:58:ILE:HD13	1:A:68:CYS:HB2	1.83	0.59
5:E:154:LYS:HG2	5:E:166:VAL:HG22	1.83	0.59
3:C:193:ILE:HG12	3:C:206[A]:ARG:HD3	1.84	0.59
3:C:282:HIS:HE1	3:C:284:VAL:HB	1.67	0.59
4:D:163:LYS:NZ	6:F:213:LYS:O	2.23	0.59
4:D:167:PRO:HD2	4:D:186:HIS:HB2	1.84	0.59
1:A:41:SER:OG	2:B:204:ARG:NH2	2.29	0.59
7:G:291:VAL:HB	7:G:318:ILE:HB	1.85	0.58
1:A:66:VAL:HG23	1:A:89:VAL:HG21	1.85	0.58
7:G:132:THR:OG1	7:G:134:HIS:NE2	2.37	0.58
3:C:316:GLN:HB2	3:C:317:PRO:HD2	1.84	0.58
3:C:31:ASP:OD1	3:C:356:ARG:NH1	2.37	0.57
7:G:239:ARG:NH1	9:K:61:ASP:OD2	2.37	0.57
1:A:282:HIS:NE2	1:A:284:VAL:HG22	2.19	0.57
3:C:328:ASN:CG	3:C:359:ALA:HB2	2.25	0.57
4:D:198:LYS:NZ	4:D:206:GLU:OE2	2.38	0.57
7:G:83:GLN:HG2	7:G:113:TRP:CE2	2.40	0.57

Continued on next page...

PRELIMINARY VALIDATION REPORT

Continued from previous page...

Atom-1	Atom-2	Interatomic distance (Å)	Clash overlap (Å)
1:A:205:ARG:HG2	1:A:207:ILE:HD13	1.87	0.57
5:E:106:CYS:HA	5:E:129:PRO:HB3	1.85	0.56
5:E:133:ILE:HD12	5:E:146:THR:HG22	1.86	0.56
1:A:40:ASP:HB2	1:A:63:ALA:HB2	1.86	0.56
5:E:252:LYS:O	5:E:252:LYS:HD3	2.05	0.56
5:E:351:ASN:OD1	11:S:20:HOH:O	2.17	0.56
1:A:239:ARG:NH1	9:X:61:ASP:OD2	2.35	0.56
5:E:112:MET:O	5:E:120:ALA:HA	2.06	0.56
5:E:340:LYS:HG2	6:F:164:PHE:CE2	2.41	0.56
7:G:100:LYS:HD2	7:G:112:MET:SD	2.46	0.55
5:E:56:PHE:CZ	5:E:70:GLU:HG3	2.41	0.55
3:C:337:ASP:OD1	3:C:339:SER:OG	2.21	0.55
8:H:182:ILE:HG22	8:H:183:SER:N	2.14	0.55
3:C:328:ASN:CB	3:C:359:ALA:HB2	2.36	0.55
5:E:214:GLN:HB3	5:E:236:ILE:HD13	1.88	0.55
7:G:29:MET:O	7:G:30:LYS:HG2	2.06	0.55
7:G:32:ILE:HG21	7:G:75:GLY:O	2.07	0.55
3:C:294:ASP:OD1	3:C:294:ASP:N	2.38	0.55
7:G:29:MET:HE3	7:G:311:SER:HA	1.88	0.55
7:G:66:VAL:HG13	7:G:89:VAL:HG21	1.89	0.55
7:G:76:GLN:HG3	7:G:77:THR:N	2.22	0.55
3:C:190:ARG:NH1	3:C:211:LEU:O	2.36	0.54
3:C:365:ARG:HD2	3:C:365:ARG:N	2.22	0.54
7:G:216:ARG:NH2	8:H:200:LEU:HB2	2.22	0.54
7:G:157:ASP:O	7:G:158:THR:OG1	2.20	0.54
5:E:282:HIS:CE1	5:E:284:VAL:HG13	2.43	0.54
3:C:274:TYR:HB3	3:C:293:SER:HB3	1.89	0.54
5:E:144:VAL:O	5:E:155:PHE:HA	2.07	0.54
5:E:157:ASP:HB2	5:E:159:ARG:HG2	1.89	0.54
7:G:300:TRP:CZ3	7:G:307:LYS:HB2	2.43	0.54
7:G:190:ARG:HA	7:G:215:HIS:CE1	2.43	0.53
3:C:307:LYS:NZ	3:C:310:THR:OG1	2.30	0.53
7:G:261:ARG:HG2	7:G:273:ILE:HG23	1.90	0.53
3:C:192:LEU:O	3:C:206[A]:ARG:HD2	2.08	0.53
8:H:202:GLU:O	8:H:206:GLU:HG3	2.09	0.53
1:A:356:ARG:NH2	1:A:361:GLU:OE2	2.41	0.53
5:E:92:VAL:HG12	5:E:103:THR:HA	1.90	0.53
7:G:106:CYS:HA	7:G:129:PRO:HB3	1.90	0.53
7:G:85:HIS:CE1	7:G:111:LYS:HG3	2.44	0.52
1:A:46:SER:HB2	1:A:92:VAL:HG23	1.91	0.52
5:E:180:TYR:CD2	5:E:181:PRO:HA	2.44	0.52

Continued on next page...

PRELIMINARY VALIDATION REPORT

Continued from previous page...

Atom-1	Atom-2	Interatomic distance (Å)	Clash overlap (Å)
1:A:183:ALA:HB3	1:A:195:TYR:HB2	1.91	0.52
3:C:42:ILE:HD12	3:C:333:ALA:HB1	1.92	0.52
3:C:106:CYS:HA	3:C:129:PRO:HB3	1.91	0.52
5:E:112:MET:HE2	5:E:123:ILE:HG21	1.92	0.52
8:H:189:ILE:O	8:H:192:MET:HB2	2.10	0.52
7:G:85:HIS:NE2	7:G:103:THR:OG1	2.30	0.52
1:A:309:LYS:HD2	9:X:53:ASP:HB2	1.90	0.51
3:C:58:ILE:HD11	3:C:94:TRP:CZ2	2.44	0.51
7:G:58:ILE:HD13	7:G:94:TRP:CZ2	2.44	0.51
7:G:62:TRP:CZ3	8:H:204:ARG:HG2	2.44	0.51
1:A:174:TYR:CZ	1:A:188:ALA:HB2	2.45	0.51
7:G:81:ALA:HB1	7:G:118:ASN:OD1	2.10	0.51
7:G:211:LEU:HD13	7:G:235:SER:HB3	1.93	0.51
7:G:180:TYR:CD2	7:G:181:PRO:HA	2.46	0.51
1:A:144:VAL:O	1:A:155:PHE:HA	2.10	0.51
1:A:211:LEU:HD13	1:A:235:SER:HB3	1.92	0.51
3:C:308:LEU:HD23	9:I:55:GLU:HG3	1.92	0.51
1:A:171:GLU:OE2	1:A:172:ARG:NH1	2.44	0.51
1:A:217:CYS:SG	1:A:279:ILE:HG13	2.51	0.51
8:H:182:ILE:CG2	8:H:183:SER:H	2.14	0.51
7:G:29:MET:CE	7:G:312:GLU:H	2.24	0.50
5:E:258:LYS:NZ	6:F:172:THR:O	2.45	0.50
1:A:190:ARG:HD3	1:A:212:LYS:HA	1.94	0.50
3:C:26:HIS:HA	3:C:315:ASP:OD1	2.12	0.49
5:E:224:LYS:HD3	5:E:224:LYS:N	2.27	0.49
5:E:272:ASP:HB3	6:F:187:GLN:HG3	1.94	0.49
2:B:198:LYS:NZ	2:B:206:GLU:OE2	2.42	0.49
7:G:31:ASP:CG	7:G:356:ARG:HG3	2.32	0.49
7:G:184:VAL:HG12	7:G:220:ILE:HD11	1.94	0.49
2:B:193:LYS:HD2	2:B:193:LYS:O	2.11	0.49
7:G:26:HIS:ND1	7:G:27:ASN:N	2.61	0.49
8:H:159:GLY:HA3	8:H:212:ARG:NH1	2.28	0.49
3:C:72:GLN:HB3	3:C:76:GLN:O	2.13	0.49
5:E:104:ALA:HB1	5:E:130:VAL:HG12	1.94	0.49
7:G:62:TRP:HA	7:G:88:PRO:HB3	1.94	0.49
1:A:85:HIS:NE2	1:A:103:THR:OG1	2.38	0.48
7:G:165:MET:HE1	7:G:201:PRO:CD	2.39	0.48
7:G:196:GLN:O	7:G:197:LEU:HD23	2.12	0.48
5:E:358:ALA:HB1	5:E:361:GLU:HB2	1.95	0.48
1:A:31:ASP:OD2	1:A:356:ARG:HD2	2.14	0.48
3:C:225:GLN:N	3:C:225:GLN:OE1	2.46	0.48

Continued on next page...

PRELIMINARY VALIDATION REPORT

Continued from previous page...

Atom-1	Atom-2	Interatomic distance (Å)	Clash overlap (Å)
1:A:221:PHE:CD1	1:A:302:LYS:HE2	2.49	0.48
1:A:282:HIS:CD2	1:A:284:VAL:H	2.30	0.48
7:G:216:ARG:NH2	8:H:201:GLU:OE2	2.47	0.48
3:C:291:VAL:HG11	3:C:321:CYS:HB3	1.96	0.48
1:A:348:GLN:O	1:A:350:LYS:NZ	2.43	0.48
1:A:41:SER:HB3	1:A:335:SER:HB3	1.96	0.48
5:E:252:LYS:HD3	5:E:252:LYS:C	2.35	0.48
7:G:69:TRP:HB3	7:G:77:THR:HB	1.95	0.48
5:E:128:ALA:HB1	5:E:129:PRO:HD2	1.96	0.47
7:G:237:GLU:O	7:G:239:ARG:N	2.47	0.47
1:A:62:TRP:HA	1:A:88:PRO:HB3	1.96	0.47
3:C:82:GLN:HG2	3:C:83:GLN:N	2.30	0.47
3:C:144:VAL:O	3:C:155:PHE:HA	2.14	0.47
1:A:26:HIS:HD2	1:A:352:TYR:CZ	2.32	0.47
1:A:282:HIS:HD2	1:A:284:VAL:H	1.61	0.47
3:C:282:HIS:CD2	3:C:327:GLY:O	2.67	0.47
5:E:62:TRP:HA	5:E:88:PRO:HB3	1.95	0.47
3:C:282:HIS:CE1	3:C:284:VAL:HB	2.48	0.47
3:C:306:THR:HB	9:I:55:GLU:HG2	1.97	0.47
3:C:180:TYR:CD2	3:C:181:PRO:HA	2.50	0.47
5:E:140:ASN:OD1	5:E:140:ASN:N	2.48	0.47
3:C:336:TYR:CE2	3:C:338:TRP:HA	2.50	0.47
5:E:113:TRP:HA	5:E:119:GLN:O	2.15	0.47
1:A:300:TRP:CZ3	1:A:307:LYS:HG3	2.50	0.46
5:E:91:ASP:HB3	5:E:133:ILE:HG22	1.97	0.46
1:A:126:HIS:CD2	1:A:130:VAL:HG22	2.50	0.46
3:C:324:ASN:ND2	3:C:326:ASN:OD1	2.48	0.46
3:C:343:GLU:HG2	5:E:346:ASN:OD1	2.15	0.46
5:E:58:ILE:HD13	5:E:94:TRP:CZ2	2.50	0.46
7:G:260:HIS:ND1	7:G:294:ASP:OD1	2.46	0.46
7:G:293:SER:HB3	7:G:337:ASP:OD2	2.15	0.46
7:G:37:SER:HA	7:G:69:TRP:HZ2	1.79	0.46
1:A:145:MET:HG3	1:A:155:PHE:CE2	2.50	0.46
2:B:204:ARG:NH1	11:S:4:HOH:O	2.37	0.46
1:A:221:PHE:HB3	1:A:281:PHE:CE2	2.50	0.46
3:C:331:ALA:HA	3:C:354:PHE:O	2.16	0.46
5:E:96:ASP:OD1	5:E:135:TRP:NE1	2.49	0.46
5:E:306:THR:HB	10:J:55:GLU:HG3	1.97	0.46
3:C:358:ALA:HB1	3:C:361:GLU:HB2	1.98	0.46
3:C:38:PRO:HA	3:C:67:ARG:NH1	2.31	0.45
7:G:255:PHE:CD2	9:K:60:ILE:HG12	2.51	0.45

Continued on next page...

PRELIMINARY VALIDATION REPORT

Continued from previous page...

Atom-1	Atom-2	Interatomic distance (Å)	Clash overlap (Å)
5:E:274:TYR:CD1	5:E:294:ASP:HB3	2.51	0.45
7:G:72:GLN:CD	7:G:78:ILE:HD11	2.36	0.45
7:G:96:ASP:OD1	7:G:137:LYS:HD3	2.16	0.45
1:A:110:ALA:HB3	1:A:124:ALA:HB3	1.99	0.45
1:A:348:GLN:C	1:A:350:LYS:HZ3	2.20	0.45
5:E:101:VAL:CG2	5:E:115:LEU:HD21	2.46	0.45
1:A:34:VAL:HG13	1:A:77:THR:HG21	1.99	0.45
1:A:112:MET:O	1:A:120:ALA:HA	2.16	0.45
1:A:126:HIS:CE1	1:A:154:LYS:HD2	2.52	0.45
3:C:258:LYS:NZ	9:I:61:ASP:OD1	2.50	0.45
5:E:41:SER:OG	6:F:204:ARG:NH2	2.40	0.45
1:A:52:LEU:HD23	1:A:52:LEU:HA	1.83	0.45
8:H:198:LYS:NZ	8:H:206:GLU:OE2	2.43	0.45
1:A:51:THR:OG1	1:A:96:ASP:O	2.19	0.44
7:G:141:TYR:OH	7:G:201:PRO:HD3	2.17	0.44
1:A:67:ARG:HB2	1:A:69:TRP:CH2	2.51	0.44
5:E:65:ASP:OD2	5:E:67:ARG:NE	2.50	0.44
1:A:65:ASP:HB3	1:A:84:MET:HE2	1.99	0.44
2:B:167:PRO:HD2	2:B:186:HIS:HB2	1.99	0.44
7:G:315:ASP:OD1	7:G:315:ASP:N	2.47	0.44
3:C:359:ALA:O	3:C:360:GLU:C	2.55	0.44
5:E:119:GLN:HA	5:E:119:GLN:OE1	2.17	0.44
7:G:34:VAL:HG23	7:G:353:ILE:O	2.16	0.44
8:H:189:ILE:HG23	8:H:190:THR:HG23	1.99	0.44
3:C:274:TYR:CD1	3:C:294:ASP:HA	2.52	0.44
4:D:205:LEU:O	4:D:209:GLN:HG3	2.18	0.44
7:G:182:MET:HE1	7:G:245:ILE:HG13	1.98	0.44
1:A:338:TRP:CD2	2:B:204:ARG:HG3	2.53	0.44
7:G:80:LYS:HD3	7:G:80:LYS:HA	1.72	0.44
8:H:164:PHE:HD1	8:H:189:ILE:HD12	1.83	0.44
7:G:211:LEU:HD21	7:G:241:ALA:HB2	1.99	0.43
5:E:336:TYR:CE2	5:E:338:TRP:HA	2.52	0.43
3:C:194:VAL:HG23	3:C:204:PHE:HB3	2.00	0.43
3:C:295:GLY:O	3:C:314:LEU:HD23	2.17	0.43
1:A:34:VAL:HG23	1:A:353:ILE:O	2.18	0.43
5:E:32:ILE:HD12	5:E:32:ILE:N	2.33	0.43
7:G:118:ASN:O	7:G:118:ASN:ND2	2.48	0.43
3:C:343:GLU:HG2	5:E:346:ASN:CG	2.39	0.43
5:E:32:ILE:HD12	5:E:32:ILE:H	1.84	0.43
7:G:298:SER:OG	7:G:310:THR:HG23	2.19	0.43
1:A:170:PRO:HG3	1:A:193:ILE:HD11	2.01	0.43

Continued on next page...

PRELIMINARY VALIDATION REPORT

Continued from previous page...

Atom-1	Atom-2	Interatomic distance (Å)	Clash overlap (Å)
6:F:164:PHE:CD1	6:F:189:ILE:HG13	2.54	0.43
3:C:35:THR:HG23	3:C:77:THR:HG23	2.00	0.43
1:A:184:VAL:HG23	1:A:220:ILE:HD11	2.01	0.43
5:E:331:ALA:HA	5:E:354:PHE:O	2.19	0.43
1:A:56:PHE:CE2	1:A:70:GLU:HB2	2.53	0.42
5:E:293:SER:HA	5:E:317:PRO:HB3	2.01	0.42
7:G:112:MET:CE	7:G:158:THR:HB	2.43	0.42
3:C:295:GLY:HA2	3:C:318:ILE:HG13	2.01	0.42
1:A:128:ALA:HB3	1:A:150:ASP:N	2.35	0.42
5:E:102:PHE:CE1	5:E:112:MET:HG3	2.55	0.42
1:A:174:TYR:CZ	2:B:199:SER:HB3	2.54	0.42
7:G:26:HIS:ND1	7:G:27:ASN:HB3	2.34	0.42
7:G:31:ASP:OD2	7:G:356:ARG:HG3	2.19	0.42
7:G:58:ILE:HD13	7:G:94:TRP:HZ2	1.84	0.42
7:G:88:PRO:HD2	7:G:106:CYS:HB2	2.02	0.42
1:A:48:SER:HB2	1:A:52:LEU:HD12	2.01	0.42
5:E:41:SER:HB3	5:E:335:SER:HB3	2.01	0.42
1:A:91:ASP:HB3	1:A:133:ILE:HG22	2.02	0.42
1:A:307:LYS:HE3	1:A:307:LYS:HB3	1.79	0.42
5:E:48:SER:HB2	5:E:52:LEU:CD1	2.49	0.42
5:E:307:LYS:HE3	5:E:310:THR:OG1	2.20	0.42
5:E:58:ILE:HD13	5:E:94:TRP:HZ2	1.85	0.41
7:G:78:ILE:HB	7:G:80:LYS:NZ	2.35	0.41
7:G:102:PHE:CE1	7:G:112:MET:HB2	2.55	0.41
7:G:112:MET:HB3	7:G:123:ILE:HD13	2.01	0.41
7:G:183:ALA:HB3	7:G:195:TYR:HB2	2.02	0.41
3:C:297:PHE:CZ	3:C:311:SER:HB3	2.55	0.41
1:A:104:ALA:HB1	1:A:130:VAL:HG12	2.03	0.41
1:A:365:ARG:NH2	11:S:65:HOH:O	2.54	0.41
3:C:187:THR:OG1	3:C:191:GLY:HA3	2.20	0.41
4:D:158:THR:OG1	4:D:159:GLY:N	2.53	0.41
5:E:272:ASP:HB3	6:F:187:GLN:CG	2.49	0.41
7:G:294:ASP:OD2	7:G:296:ARG:HG2	2.20	0.41
5:E:49:PRO:HD2	5:E:52:LEU:HD12	2.02	0.41
7:G:187:THR:OG1	7:G:191:GLY:HA3	2.21	0.41
7:G:362:LEU:HD23	7:G:362:LEU:HA	1.89	0.41
1:A:200:GLN:HE21	1:A:200:GLN:HB2	1.71	0.41
3:C:192:LEU:HD12	3:C:192:LEU:HA	1.93	0.41
5:E:324:ASN:ND2	5:E:326:ASN:OD1	2.48	0.41
7:G:31:ASP:OD1	7:G:356:ARG:HG3	2.21	0.41
7:G:216:ARG:HH21	8:H:200:LEU:HB2	1.83	0.41

Continued on next page...

PRELIMINARY VALIDATION REPORT

Continued from previous page...

Atom-1	Atom-2	Interatomic distance (Å)	Clash overlap (Å)
1:A:190:ARG:HD2	1:A:211:LEU:O	2.21	0.41
1:A:211:LEU:HD23	1:A:211:LEU:HA	1.83	0.41
2:B:201:GLU:OE1	2:B:201:GLU:N	2.46	0.41
5:E:211:LEU:HD13	5:E:235:SER:HB3	2.02	0.41
7:G:288:LEU:HG	7:G:300:TRP:HB2	2.02	0.41
1:A:256:THR:OG1	9:X:61:ASP:HB2	2.21	0.41
5:E:26:HIS:HA	5:E:315:ASP:OD1	2.20	0.41
5:E:178:VAL:HG22	5:E:183:ALA:HB2	2.03	0.40
5:E:183:ALA:HB3	5:E:195:TYR:HB2	2.03	0.40
5:E:282:HIS:HE1	5:E:284:VAL:HG13	1.86	0.40
1:A:221:PHE:HB3	1:A:281:PHE:CZ	2.57	0.40
3:C:106:CYS:SG	4:D:205:LEU:HD13	2.62	0.40
7:G:76:GLN:CG	7:G:77:THR:N	2.84	0.40
3:C:211:LEU:HD12	3:C:233:LEU:HD23	2.03	0.40
3:C:217:CYS:SG	3:C:279:ILE:HG13	2.62	0.40
3:C:261:ARG:HG2	3:C:273:ILE:HA	2.02	0.40

There are no symmetry-related clashes.

5.3 Torsion angles (i)

5.3.1 Protein backbone (i)

In the following table, the Percentiles column shows the percent Ramachandran outliers of the chain as a percentile score with respect to all X-ray entries followed by that with respect to entries of similar resolution.

The Analysed column shows the number of residues for which the backbone conformation was analysed, and the total number of residues.

Mol	Chain	Analysed	Favoured	Allowed	Outliers	Percentiles	
1	A	335/339 (99%)	327 (98%)	8 (2%)	0	100	100
2	B	47/51 (92%)	44 (94%)	2 (4%)	1 (2%)	7	23
3	C	334/337 (99%)	323 (97%)	11 (3%)	0	100	100
4	D	40/44 (91%)	38 (95%)	2 (5%)	0	100	100
5	E	330/336 (98%)	324 (98%)	6 (2%)	0	100	100
6	F	50/54 (93%)	48 (96%)	2 (4%)	0	100	100
7	G	331/336 (98%)	327 (99%)	4 (1%)	0	100	100

Continued on next page...

PRELIMINARY VALIDATION REPORT

Continued from previous page...

Mol	Chain	Analysed	Favoured	Allowed	Outliers	Percentiles	
8	H	42/46 (91%)	40 (95%)	2 (5%)	0	100	100
9	I	7/9 (78%)	7 (100%)	0	0	100	100
9	K	7/9 (78%)	7 (100%)	0	0	100	100
9	X	7/9 (78%)	7 (100%)	0	0	100	100
10	J	6/8 (75%)	6 (100%)	0	0	100	100
All	All	1536/1578 (97%)	1498 (98%)	37 (2%)	1 (0%)	51	81

All (1) Ramachandran outliers are listed below:

Mol	Chain	Res	Type
2	B	193	LYS

5.3.2 Protein sidechains ⓘ

In the following table, the Percentiles column shows the percent sidechain outliers of the chain as a percentile score with respect to all X-ray entries followed by that with respect to entries of similar resolution.

The Analysed column shows the number of residues for which the sidechain conformation was analysed, and the total number of residues.

Mol	Chain	Analysed	Rotameric	Outliers	Percentiles	
1	A	295/295 (100%)	282 (96%)	13 (4%)	28	61
2	B	47/47 (100%)	45 (96%)	2 (4%)	29	62
3	C	294/293 (100%)	275 (94%)	19 (6%)	17	44
4	D	40/40 (100%)	38 (95%)	2 (5%)	24	56
5	E	292/292 (100%)	278 (95%)	14 (5%)	25	58
6	F	49/49 (100%)	46 (94%)	3 (6%)	18	48
7	G	293/293 (100%)	266 (91%)	27 (9%)	9	27
8	H	42/42 (100%)	40 (95%)	2 (5%)	25	58
9	I	9/9 (100%)	8 (89%)	1 (11%)	6	19
9	K	9/9 (100%)	8 (89%)	1 (11%)	6	19
9	X	9/9 (100%)	9 (100%)	0	100	100
10	J	8/8 (100%)	8 (100%)	0	100	100
All	All	1387/1386 (100%)	1303 (94%)	84 (6%)	18	48

PRELIMINARY VALIDATION REPORT

All (84) residues with a non-rotameric sidechain are listed below:

Mol	Chain	Res	Type
1	A	26	HIS
1	A	28	PRO
1	A	41	SER
1	A	72	GLN
1	A	86	THR
1	A	148	SER
1	A	161	SER
1	A	200	GLN
1	A	215	HIS
1	A	252	LYS
1	A	315	ASP
1	A	324	ASN
1	A	338	TRP
2	B	171	ASP
2	B	211	ASN
3	C	37	SER
3	C	61	SER
3	C	64	ASN
3	C	67	ARG
3	C	71	VAL
3	C	72	GLN
3	C	73	ASP
3	C	77	THR
3	C	112	MET
3	C	165	MET
3	C	171	GLU
3	C	227	LYS
3	C	274	TYR
3	C	302	LYS
3	C	328	ASN
3	C	338	TRP
3	C	349	LYS
3	C	352	TYR
3	C	365	ARG
4	D	161	THR
4	D	212	ARG
5	E	47	PHE
5	E	67	ARG
5	E	105	SER
5	E	112	MET
5	E	148	SER
5	E	171	GLU

Continued on next page...

PRELIMINARY VALIDATION REPORT

Continued from previous page...

Mol	Chain	Res	Type
5	E	200	GLN
5	E	224	LYS
5	E	268	SER
5	E	293	SER
5	E	302	LYS
5	E	338	TRP
5	E	360	GLU
5	E	365	ARG
6	F	171	ASP
6	F	180	THR
6	F	183	SER
7	G	30	LYS
7	G	33	GLU
7	G	47	PHE
7	G	67	ARG
7	G	70	GLU
7	G	76	GLN
7	G	92	VAL
7	G	109	THR
7	G	112	MET
7	G	118	ASN
7	G	121	ILE
7	G	148	SER
7	G	157	ASP
7	G	159	ARG
7	G	166	VAL
7	G	168	GLN
7	G	171	GLU
7	G	200	GLN
7	G	217	CYS
7	G	218	VAL
7	G	288	LEU
7	G	296	ARG
7	G	302	LYS
7	G	307	LYS
7	G	309	LYS
7	G	334	SER
7	G	338	TRP
8	H	168	THR
8	H	180	THR
9	I	55	GLU
9	K	55	GLU

PRELIMINARY VALIDATION REPORT

Sometimes sidechains can be flipped to improve hydrogen bonding and reduce clashes. All (6) such sidechains are listed below:

Mol	Chain	Res	Type
1	A	134	HIS
1	A	140	ASN
1	A	282	HIS
2	B	186	HIS
5	E	196	GLN
9	X	56	GLN

5.3.3 RNA (i)

There are no RNA molecules in this entry.

5.4 Non-standard residues in protein, DNA, RNA chains (i)

There are no non-standard protein/DNA/RNA residues in this entry.

5.5 Carbohydrates (i)

There are no monosaccharides in this entry.

5.6 Ligand geometry (i)

There are no ligands in this entry.

5.7 Other polymers (i)

There are no such residues in this entry.

5.8 Polymer linkage issues (i)

The following chains have linkage breaks:

Mol	Chain	Number of breaks
7	G	2
5	E	2
8	H	1
3	C	1

Continued on next page...

PRELIMINARY VALIDATION REPORT

Continued from previous page...

Mol	Chain	Number of breaks
2	B	1
1	A	1
4	D	1
6	F	1

All chain breaks are listed below:

Model	Chain	Residue-1	Atom-1	Residue-2	Atom-2	Distance (Å)
1	H	169:GLY	C	180:THR	N	12.92
1	G	263:ASN	C	268:SER	N	11.10
1	E	263:ASN	C	268:SER	N	9.38
1	C	263:ASN	C	268:SER	N	7.49
1	B	173:MET	C	179:SER	N	6.61
1	A	263:ASN	C	266:ASN	N	5.42
1	D	170:THR	C	183:SER	N	4.74
1	F	174:VAL	C	177:GLY	N	4.41
1	E	198:GLU	C	200:GLN	N	3.38
1	G	237:GLU	C	239:ARG	N	3.17

PRELIMINARY VALIDATION REPORT

6 Fit of model and data (i)

6.1 Protein, DNA and RNA chains (i)

In the following table, the column labelled '#RSRZ> 2' contains the number (and percentage) of RSRZ outliers, followed by percent RSRZ outliers for the chain as percentile scores relative to all X-ray entries and entries of similar resolution. The OWAB column contains the minimum, median, 95th percentile and maximum values of the occupancy-weighted average B-factor per residue. The column labelled 'Q< 0.9' lists the number of (and percentage) of residues with an average occupancy less than 0.9.

Mol	Chain	Analysed	<RSRZ>	#RSRZ>2			OWAB(Å^2)	Q<0.9
1	A	339/339 (100%)	-0.25	0	100	100	35, 50, 80, 123	0
2	B	51/51 (100%)	-0.01	2 (3%)	39	29	44, 60, 102, 133	0
3	C	337/337 (100%)	-0.20	4 (1%)	79	73	47, 77, 116, 151	0
4	D	44/44 (100%)	0.03	0	100	100	58, 84, 98, 116	0
5	E	336/336 (100%)	-0.32	1 (0%)	94	93	42, 66, 102, 138	0
6	F	54/54 (100%)	-0.44	0	100	100	49, 64, 96, 106	0
7	G	336/336 (100%)	0.01	6 (1%)	68	61	47, 81, 117, 154	0
8	H	46/46 (100%)	0.58	5 (10%)	5	3	77, 113, 171, 180	0
9	I	9/9 (100%)	0.01	0	100	100	82, 93, 138, 144	0
9	K	9/9 (100%)	1.61	3 (33%)	0	0	81, 88, 128, 129	0
9	X	9/9 (100%)	0.84	3 (33%)	0	0	60, 73, 111, 126	0
10	J	8/8 (100%)	-0.11	0	100	100	60, 68, 99, 112	0
All	All	1578/1578 (100%)	-0.15	24 (1%)	73	68	35, 69, 116, 180	0

All (24) RSRZ outliers are listed below:

Mol	Chain	Res	Type	RSRZ
9	K	54	GLU	4.6
7	G	263	ASN	4.4
9	K	53	ASP	4.2
3	C	360	GLU	3.6
3	C	270	PRO	2.9
8	H	180	THR	2.7
8	H	182	ILE	2.7
2	B	179	SER	2.7
3	C	263	ASN	2.5
8	H	161	THR	2.5
9	K	55	GLU	2.4

Continued on next page...

PRELIMINARY VALIDATION REPORT

Continued from previous page...

Mol	Chain	Res	Type	RSRZ
8	H	195	TYR	2.4
9	X	53	ASP	2.4
5	E	365	ARG	2.4
8	H	183	SER	2.3
7	G	53	PRO	2.3
7	G	366	ASN	2.3
9	X	56	GLN	2.2
7	G	26	HIS	2.2
7	G	54	GLY	2.1
9	X	54	GLU	2.1
3	C	315	ASP	2.0
7	G	113	TRP	2.0
2	B	161	THR	2.0

6.2 Non-standard residues in protein, DNA, RNA chains (i)

There are no non-standard protein/DNA/RNA residues in this entry.

6.3 Carbohydrates (i)

There are no monosaccharides in this entry.

6.4 Ligands (i)

There are no ligands in this entry.

6.5 Other polymers (i)

There are no such residues in this entry.

PRELIMINARY VALIDATION REPORT
