# Supplementary material for: Molecular Mechanism of SARS-CoVs Orf6 Targeting the Rae1–Nup98 Complex to Compete With mRNA Nuclear Export
Source: Front Mol Biosci. 2022 Jan 12;8:813248. doi: 10.3389/fmolb.2021.813248 (PMC8790125; doi:10.3389/fmolb.2021.813248)
Supplement: Supplementary file 2 [file DataSheet4.docx]

Supplementary Material for

Molecular mechanism of SARS-CoVs Orf6 targeting the Rae1–Nup98 complex to compete with mRNA nuclear export

# Supplementary Figures


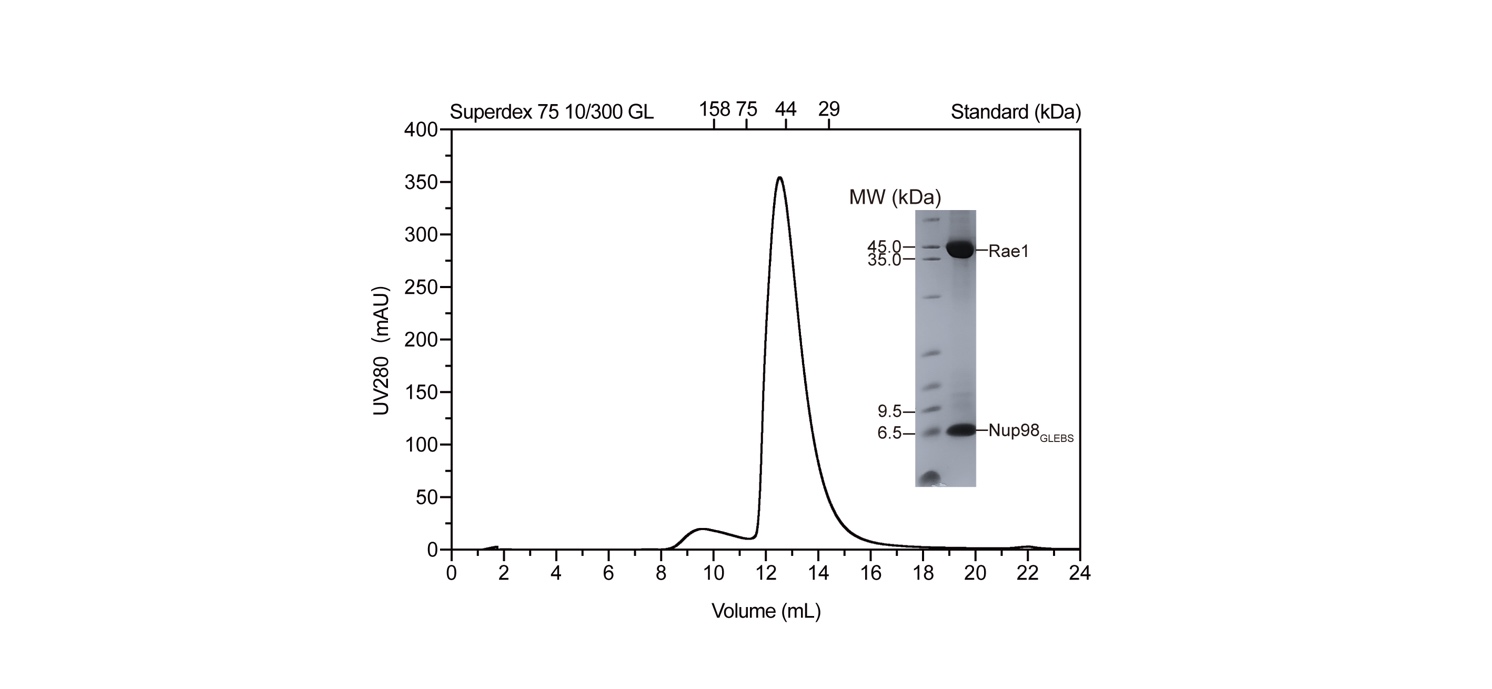


**Figure S1.** **Recombinant protein purification of the Rae1–Nup98_GLEBS_ complex.** The size exclusion chromatograms of the Rae1–Nup98_GLEBS_ complex (Superdex 75 Increase 10/300 GL). The molecular masses of protein standards are indicated at the top. Protein sample was examined on a 16% Tricine–SDS-polyacrylamide gel (Schagger, H., Nat Protoc, 2006). The theoretical molecular weight of each protein: Rae1 (42340 Da), Nup98_GLEBS_ (7767 Da).


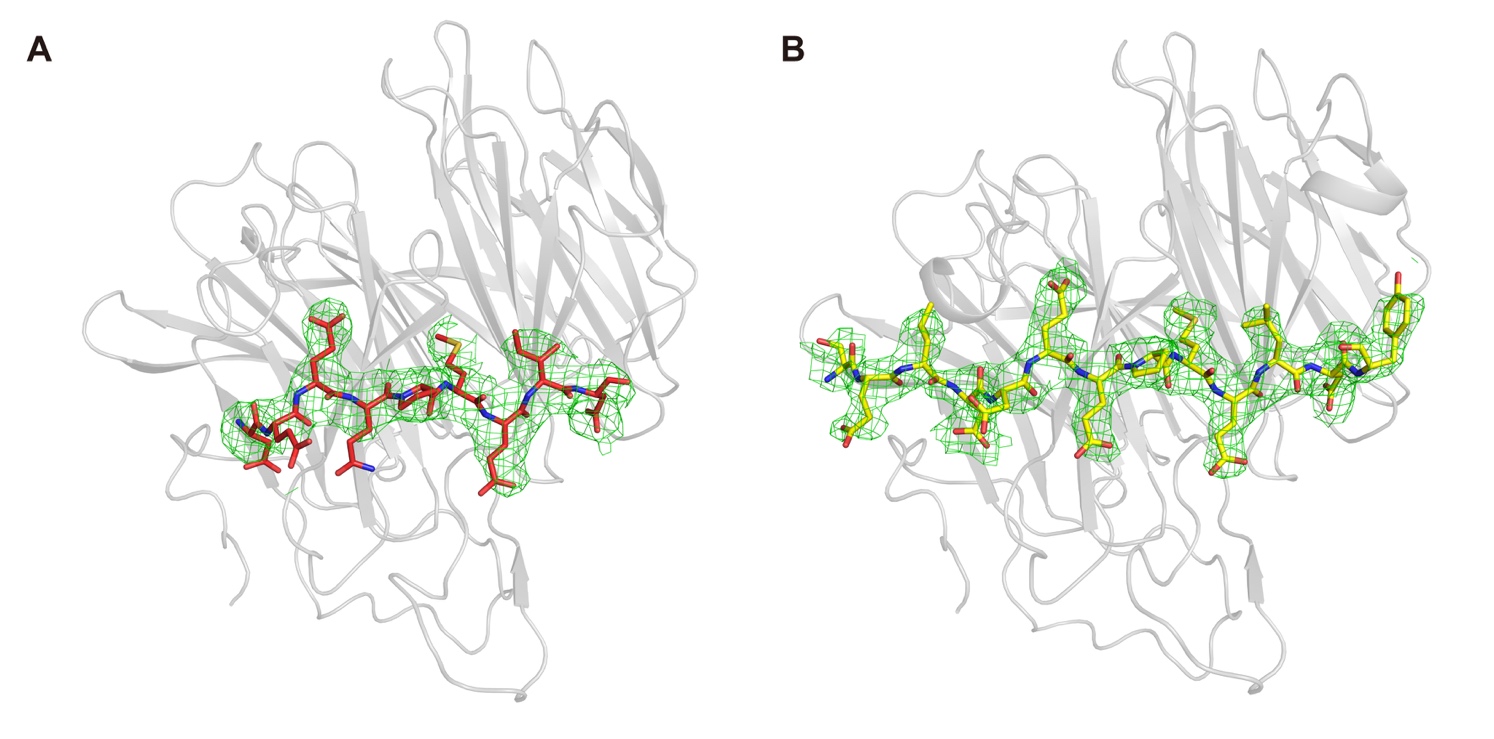


**Figure S2.** **Electron density maps for the SARS-CoVs Orf6_CTT_ peptides inside the hydrophobic pocket of Rae1**. The omit maps (green mesh, calculated using program phenix.composite_omit_map) for SARS-CoV-2 Orf6_CTT_ (A) and SARS-CoV-1 Orf6_CTT_ (B) are superimposed on the model.


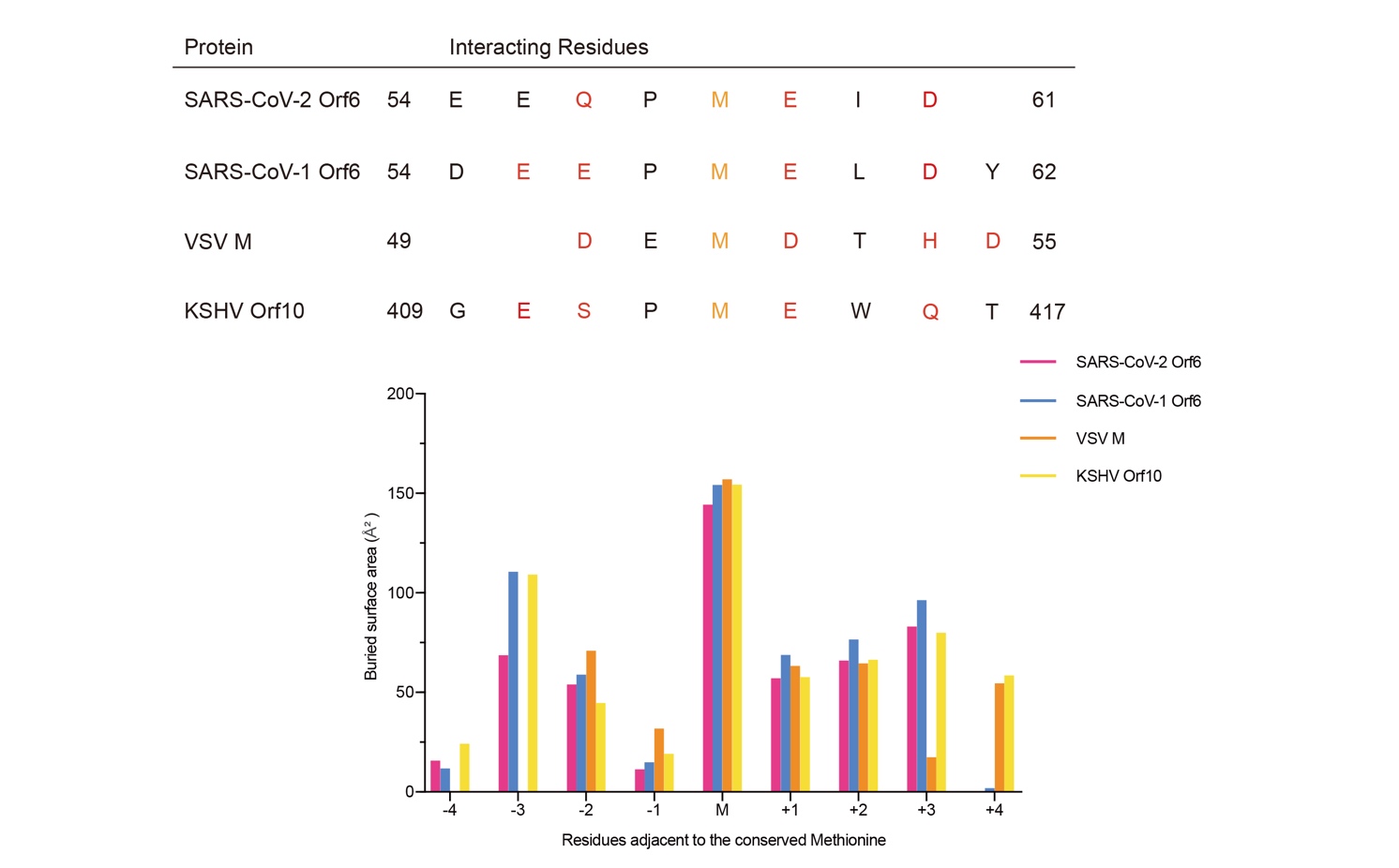


**Figure S3.** **M58 of SARS-CoVs Orf6_CTT_ is critical for the interactions with the Rae1–Nup98_GLEBS_ complex.** Sequence alignment and buried surface areas comparison of the Rae1–Nup98 interaction motifs on SARS-CoVs Orf6, VSV M and KSHV Orf10. The conserved methionine is colored in yellow and the surrounding residues which participate in interactions with the Rae1–Nup98_GLEBS_ complex are colored in red. The interface areas were calculated using PDBePISA tool (http://www.ebi.ac.uk/pdbe/pisa/) (Krissinel, E., Henrick, K., J Mol Biol, 2007).

**Figure S4. Sequence alignment of Orf6 from 155 SARS-CoV-2 variants.** Sequences were fetched from the Uniprot database. The high sequence conservation (>95%) at each position is highlighted in different colors (blue for hydrophobic amino acids, magenta for negative charged amino acids, red for positive charged amino acids, green for polar amino acids, cyan for aromatic amino acids and yellow for prolines).


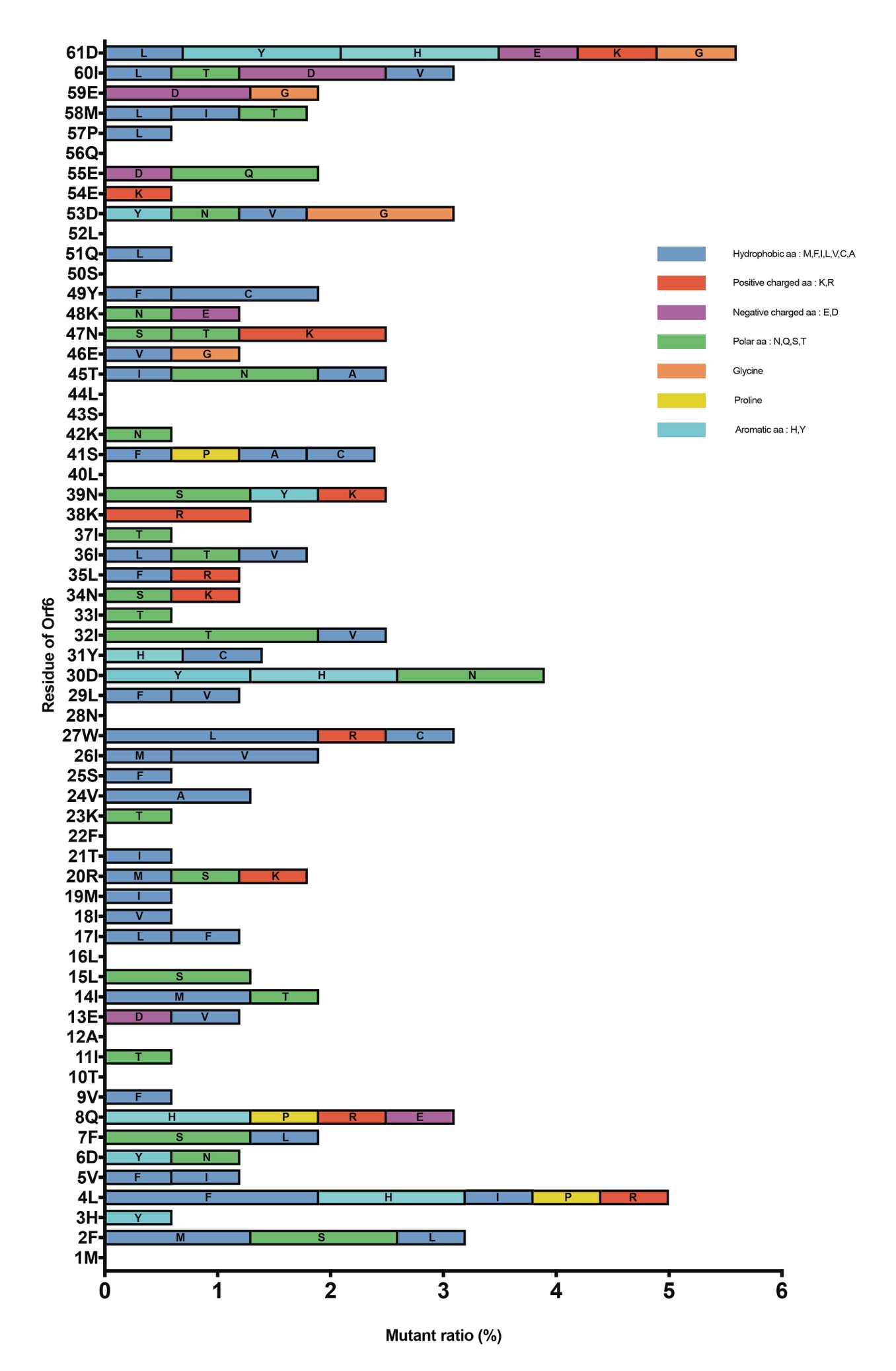


**Figure S5. Mutational profile of SARS-CoV-2 Orf6.** Relative probability of each amino acid change for mutations across 155 sequences obtained from Uniprot in the study.

# Supplementary Tables

**Table S1. Data collection and refinement statistics.**

| Data collection | SARS-CoV-2 Orf6_CTT_–Rae1–Nup98_GLEBS_ | SARS-CoV-1 Orf6_CTT_–Rae1–Nup98_GLEBS_ |
| --- | --- | --- |
| **Data Collection** |  |  |
| Space group | *C*2 | *C*2 |
| Cell dimensions |  |  |
| a, b, c (Å) | 150.66, 103.32, 136.59 | 149.61, 103.30, 134.94 |
| α, β, γ (°) | 90.0, 97.5, 90.0 | 90.0, 96.0, 90.0 |
| Resolution range* (Å) | 50.0-2.80 (2.85-2.80) | 48.2-2.49 (2.57-2.49) |
| Unique reflections | 51228 (5104) | 71539 (7151) |
| Completeness (%) | 99.48 (99.61) | 99.74 (99.93) |
| Redundancy | 3.3 | 4.4 |
| *I*/σ*I* | 10.1 (1.0) | 7.6 (1.4) |
| *R*_merge_ | 0.159 (1.549) | 0.188 (1.178) |
| CC_1/2_ | 0.99 (0.58) | 0.99 (0.62) |
|  |  |  |
| **Refinement** |  |  |
| Resolution range (Å) | 37.35-2.80 (2.90-2.80) | 44.7-2.49 (2.57-2.49) |
| No. of reflections (working/test) | 51064/1987 | 71527/1988 |
| *R*_work_/*R*_free_ | 0.192/0.244 | 0.186/0.229 |
| Number of atoms |  |  |
| Macromolecules | 12515 | 12729 |
| Ligand/ion | 0 | 0 |
| Water | 56 | 407 |
| B-factors |  |  |
| Macromolecules | 73.3 | 46.0 |
| Water | 50.8 | 40.1 |
| R.m.s. deviations |  |  |
| Bond lengths (Å) | 0.010 | 0.009 |
| Bond angles (°) | 1.4 | 1.2 |
| Ramachandran plot |  |  |
| Favored (%) | 97.52 | 97.70 |
| Allowed (%) | 2.41 | 2.30 |
| Outliers (%) | 0.07 | 0 |

Single crystal was used for each data collection and structure determination.

* Numbers in the parentheses are for the highest resolution shell.

**Table S2. Missense** **mutations in Orf6 from 155 SARS-CoV-2 variants**

| **UniProtKB entries** | **Mutations** | **First strain reported** | **UniProtKB entries** | **Mutations** | **First strain reported** |
| --- | --- | --- | --- | --- | --- |
| A0A7U3HJ56 | **D30Y** | hCoV-19/France/ARA-10968/2020 | A0A7U0IIW2 | **K48N** | hCoV-19/England/PHEC-1BFAB/2020 |
| A0A6N1UKI0 | **N39S** | hCoV-19/USA/WA-UW-3012/2020 | A0A7M1HPU5 | **S41P** | hCoV-19/Australia/QLD1245/2020 |
| A0A7M1G0F3 | **I26V** | hCoV-19/England/TFCI-2704676/2020 | A0A7D7CSJ2 | **S41A** | hCoV-19/USA/LA-SR0155/2020 |
| A0A7U0ISC5 | **D30H** | hCoV-19/USA/UT-UPHL-2101568216/2020 | A0A7T0QCB2 | **K23T** | hCoV-19/USA/WA-UW-3186/2020 |
| A0A6M3HPW4 | **Q8H** | hCoV-19/Beijing/Wuhan_IME-BJ07/2020 | A0A7T5EC92 | **I36T** | hCoV-19/Italy/LOM-Pavia-38131/2020 |
| A0A7S9GKV9 | **F2S** | hCoV-19/Switzerland/BS-UHB-42190622/2020 | A0A7T6Y695 | **E54K** | hCoV-19/USA/CA-LACPHL-AF00076/2020 |
| A0A7U1LXJ2 | **N47K** | hCoV-19/Denmark/DCGC-12471/2020 | A0A7U3DZY7 | **L4R** | hCoV-19/USA/CA-CZB-14886/2020 |
| A0A7T8HCB8 | **I32T**  **W27L** | hCoV-19/France/ARA-12260/2020  hCoV-19/England/20118179304/2020 | A0A7T8IHL8 | **D53N** | hCoV-19/USA/MI-iSPEC-0032/2020 |
| A0A7U1HE93 | **Y31del** | hCoV-19/USA/UT-QDX-2215/2020 | A0A7U3F072 | **I14M**  **F7S** | hCoV-19/Germany/BW-UKT-a002/2020  hCoV-19/United Arab Emirates/0318/2020 |
| A0A7D7KVN9 | **F7L** | hCoV-19/USA/WA-S1479/2020 | A0A7T1CF11 | **N34K** | hCoV-19/Spain/RI-IBV-005936/2020 |
| A0A6M4MT03 | **D6Y** | hCoV-19/Australia/VIC203/2020 | A0A7D5HRB0 | **I37T** | hCoV-19/Australia/SA0217/2020 |
| A0A6G9KDF2 | **V9F** | hCoV-19/USA/un-UW219/2020 | A0A7U3EXL8 | **S41C** | hCoV-19/Netherlands/un-EMC-725/2020 |
| A0A6M6D9X9 | **E13D** | hCoV-19/Ireland/D-NVRL-20G41469/2020 | A0A7G9KX67 | **I60L** | hCoV-19/USA/AZ-TG347316/2020 |
| A0A7L8M6C3 | **N47T** | hCoV-19/USA/MI-QDX-825/2020 | A0A7U0T253 | **E59G** | hCoV-19/Italy/LOM-Pavia-41747/2020 |
| A0A7D7Y430 | **L29V** | hCoV-19/USA/FL-BPHL-0650/2020 | A0A7D8AJG4 | **S25F** | hCoV-19/USA/NY-QDX-2453/2020 |
| A0A6M4SWE0 | **T21I** | hCoV-19/Spain/VC-FISABIO-50/2020 | A0A6M3WHL2 | **I33T** | hCoV-19/Chile/AR-265171/2020 |
| A0A7U2CMY6 | **L35F** | hCoV-19/Argentina/PAIS-G0070/2020 | A0A6H1XM06 | **K42N** | hCoV-19/USA/WA-UW44/2020 |
| A0A6M4MUN3 | **W27L** | hCoV-19/England/20118179304/2020 | A0A7T9KZF9 | **L29F** | hCoV-19/Canada/AB-30521/2020 |
| A0A7U1D8Z3 | **M58I** | hCoV-19/Australia/WA130/2020 | A0A7U1AYN4 | **R20M** | hCoV-19/Switzerland/GR-ETHZ-100143/2020 |
| A0A7T9KSV0 | **D6N** | hCoV-19/Peru/JUN-INS-153/2020 | A0A7S9DDE9 | **I17F** | hCoV-19/USA/MN-MDH-1289/2020 |
| A0A6M6D2N8 | **P57L** | hCoV-19/USA/NM-UNM-00054/2020 | A0A7L9RZ75 | **Q8R** | hCoV-19/Canada/ON-UHTC_0025/2020 |
| A0A6N0C498 | **I11T** | hCoV-19/Australia/VIC333/2020 | A0A7U3HTK4 | **Y49F** | hCoV-19/Australia/VIC1461/2020 |
| A0A7U2E085 | **T45I** | hCoV-19/Portugal/PT1309/2020 | A0A7D5EFC0 | **E46G** | hCoV-19/USA/WI-UW-65/2020 |
| A0A7T8KNG7 | **M19I** | hCoV-19/Italy/VEN-SI_139/2020 | A0A7G9XAY7 | **I32T** | hCoV-19/France/ARA-12260/2020 |
| A0A6M8F805 | **D53G** | hCoV-19/USA/CA-QDX-1986/2020 | A0A7M1GD69 | **N47S** | hCoV-19/USA/CA-QDX-554/2020 |
| A0A6M5CCW3 | **H3Y** | hCoV-19/France/IDF-2278/2020 | A0A6N0VU62 | **M58T** | hCoV-19/Russia/StPetersburg-RII5644S/2020 |
| A0A7T5QWX8 | **D61G** | - | A0A7L8K753 | **W27R** | hCoV-19/England/LIVE-A4AE2/2020 |
| A0A6H2EK71 | **N34S** | hCoV-19/Spain/CT-HUVH-VH2683/2020 | A0A6N1WNL0 | **M58L** | hCoV-19/USA/WA-S1020/2020 |
| A0A7G8KR33 | **Q8P** | hCoV-19/Philippines/PH-RITM-0074/2020 | A0A7M2YT51 | **I32V** | hCoV-19/USA/UT-UPHL-2101599771/2020 |
| A0A7D7CQW7 | **L4P** | hCoV-19/Israel/CVL-n-5908/2020 | A0A6N1NMT8 | **I18V** | hCoV-19/Australia/WA19/2020 |
| A0A7G9U4I6 | **I14T** | hCoV-19/United Arab Emirates/L3290248480/2020 | A0A7G8Z7D0 | **R20K** | hCoV-19/Italy/APU-IZSPB-67PT/2020 |
| A0A7U1BL67 | **I17L** | hCoV-19/USA/TX-HMH-MCoV-35378/2020 | A0A6M4NGE3 | **V5F** | hCoV-19/Japan/DP0724/2020 |
| A0A7L9W1S1 | **E55D** | hCoV-19/Wales/PHW27/2020 | A0A7D5QTE7 | **K48E** | hCoV-19/USA/FL-TGH-0104/2020 |
| A0A7H0BRZ4 | **S41F** | hCoV-19/Denmark/ALAB-SSI200/2020 | A0A6N1NRM2 | **N39K** | hCoV-19/USA/CA-UCI-071/2020 |
| A0A7H0BRZ4 | **F7S** | hCoV-19/United Arab Emirates/0318/2020 | A0A6M4N8I6 | **D53Y** | hCoV-19/USA/NV-NSPHL-A0014/2020 |
| A0A7U0TEA3 | **I60V** | hCoV-19/USA/MDH-8144/2020 | A0A7M1F0K3 | **Y31C** | hCoV-19/Italy/CAM-TIGEM-954/2020 |
| A0A7S9SLM5 | **I26M** | hCoV-19/USA/MN-MDH-1901/2020 | A0A7U1BAX6 | **I14M** | hCoV-19/Germany/BW-UKT-a002/2020 |
| A0A6N1WL69 | **R20S** | hCoV-19/Senegal/073/2020 | A0A7M3UVG0 | **D53V** | hCoV-19/USA/TX-DSHS-0533/2020 |
| A0A7M1F2T3 | **V5I** | hCoV-19/Japan/PG-1452/2020 | A0A7G6WD27 | **L4I** | hCoV-19/Ghana/2709_S25/2020 |
| A0A6N1WB67 | **Y31H** | hCoV-19/Japan/PG-1275/2020 | A0A7U0IGI9 | **T45A** | hCoV-19/Canada/QC-L00255941/2020 |
| A0A7M1G9Y5 | **L35R** | hCoV-19/USA/UT-UPHL-2009631/2020 | A0A6N0C4F1 | **N39Y** | hCoV-19/Bangladesh/BCSIR-NILMRC-067/2020 |
| A0A7U1QWC6 | **K38R** | hCoV-19/Netherlands/ZH-EMC-932/2020 | A0A7G5WX46 | **W27C** | hCoV-19/USA/MA-MASPHL-00301/2020 |
| A0A7D7W7U3 | **E55Q** | hCoV-19/USA/FL-BPHL-0662/2020 | A0A7U1QWC6 | **K38R** | hCoV-19/Netherlands/ZH-EMC-932/2020 |
| A0A7D7GIV7 | **L4H** | hCoV-19/USA/CA-ALSR-0657-IPL/2020 | A0A7D7W7U3 | **E55Q** | hCoV-19/USA/FL-BPHL-0662/2020 |
| A0A6M3Q9K0 | **V24A** | hCoV-19/Germany/HE-FFM1/2020 | A0A7D7GIV7 | **L4H** | hCoV-19/USA/CA-ALSR-0657-IPL/2020 |
| A0A7T6ZK66 | **L4F** | hCoV-19/England/CAMB-81A36/2020 | A0A6M3Q9K0 | **V24A** | hCoV-19/Germany/HE-FFM1/2020 |
| A0A7U0MJ13 | **L15S** | hCoV-19/England/QEUH-9C9C51/2020 | A0A7T6ZK66 | **L4F** | hCoV-19/England/CAMB-81A36/2020 |
| A0A7U3HSA9 | **T45N** | hCoV-19/USA/TX-HMH-MCoV-35101/2020 | A0A7U0MJ13 | **L15S** | hCoV-19/England/QEUH-9C9C51/2020 |
| A0A7U2GTC2 | **D30N** | hCoV-19/England/GSTT-265D3B4/2020 | A0A7U3HSA9 | **T45N** | hCoV-19/USA/TX-HMH-MCoV-35101/2020 |
| A0A6N1WK26 | **Y49C** | hCoV-19/Australia/VIC595/2020 | A0A7U2GTC2 | **D30N** | hCoV-19/England/GSTT-265D3B4/2020 |
| A0A7M1YHC3 | **E59D** | hCoV-19/England/NOTT-10E669/2020 | A0A6N1WK26 | **Y49C** | hCoV-19/Australia/VIC595/2020 |
| A0A7H4JFB2 | **I60T** | hCoV-19/USA/TX-HMH0211/2020 | A0A7M1YHC3 | **E59D** | hCoV-19/England/NOTT-10E669/2020 |
| A0A7U2CLJ7 | **Q8E** | hCoV-19/Japan/PG-24185/2020 | A0A7U3HJ56 | **D30Y** | hCoV-19/France/ARA-10968/2020 |
| A0A7T5XHZ7 | **L4F** | hCoV-19/England/CAMB-81A36/2020 | A0A6N1UKI0 | **N39S** | hCoV-19/USA/WA-UW-3012/2020 |
| A0A7U1QYE3 | **I36L** | hCoV-19/England/LIVE-A2C93/2020 | A0A7M1G0F3 | **I26V** | hCoV-19/England/TFCI-2704676/2020 |
| A0A7U3HJJ6 | **I36V** | hCoV-19/Spain/MD-H12-72/2020 | A0A7U0ISC5 | **D30H** | hCoV-19/USA/UT-UPHL-2101568216/2020 |
| A0A6M8F2P6 | **D53G** | hCoV-19/USA/CA-QDX-1986/2020 | A0A6M3HPW4 | **Q8H** | hCoV-19/Beijing/Wuhan_IME-BJ07/2020 |
| A0A7S6A9F0 | **F2L** | hCoV-19/England/20140002604/2020 | A0A7S9GKV9 | **F2S** | hCoV-19/Switzerland/BS-UHB-42190622/2020 |
| A0A7G9ZR76 | **E13V** | hCoV-19/Australia/VIC2890/2020 | A0A7U1LXJ2 | **N47K** | hCoV-19/Denmark/DCGC-12471/2020 |
| A0A6G6XEB8 | **E46V** | hCoV-19/USA/UT-UPHL-210609316756/2021 | A0A7T8HCB8 | **W27L**  **I32T** | hCoV-19/England/20118179304/2020  hCoV-19/France/ARA-12260/2020 |
| A0A7U0IDM6 | **Q51L** | hCoV-19/USA/NC-UNC-LCCC0060/2020 | A0A7U1HE93 | **Y31del** | hCoV-19/USA/UT-QDX-2215/2020 |
| A0A7T0YLS5 | **D61E** | hCoV-19/Qatar/QA-QU_05-I7/2020 |  |  |  |
