# Supplementary material for: Molecular Mechanism of SARS-CoVs Orf6 Targeting the Rae1–Nup98 Complex to Compete With mRNA Nuclear Export
Source: Front Mol Biosci. 2022 Jan 12;8:813248. doi: 10.3389/fmolb.2021.813248 (PMC8790125; doi:10.3389/fmolb.2021.813248)
Supplement: Supplementary file 4 [file DataSheet1.PDF]

WORLDWIDE PDB PROTEIN DATA BANK

Preliminary Full wwPDB X-ray Structure Validation Report (i)

Dec 8, 2021 – 09:46 PM JST

Deposition ID : D_1300025119

This is a Preliminary Full wwPDB X-ray Structure Validation Report.

This report is produced by the wwPDB Deposition System during initial deposition but before annotation of the structure.

We welcome your comments at *validation@mail.wwpdb.org*
A user guide is available at
https://www.wwpdb.org/validation/2017/XrayValidationReportHelp
with specific help available everywhere you see the (i) symbol.

PRELIMINARY VALIDATION REPORT

The following versions of software and data (see references (i)) were used in the production of this report:

MolProbity	:	4.02b-467
Xtriage (Phenix)	:	1.13
EDS	:	2.24
Percentile statistics	:	20191225.v01 (using entries in the PDB archive December 25th 2019)
Refmac	:	5.8.0158
CCP4	:	7.0.044 (Gargrove)
Ideal geometry (proteins)	:	Engh & Huber (2001)
Ideal geometry (DNA, RNA)	:	Parkinson et al. (1996)
Validation Pipeline (wwPDB-VP)	:	2.24

1 Overall quality at a glance (i)

The following experimental techniques were used to determine the structure:
X-RAY DIFFRACTION

The reported resolution of this entry is 2.49 Å.

Percentile scores (ranging between 0-100) for global validation metrics of the entry are shown in the following graphic. The table shows the number of entries on which the scores are based.

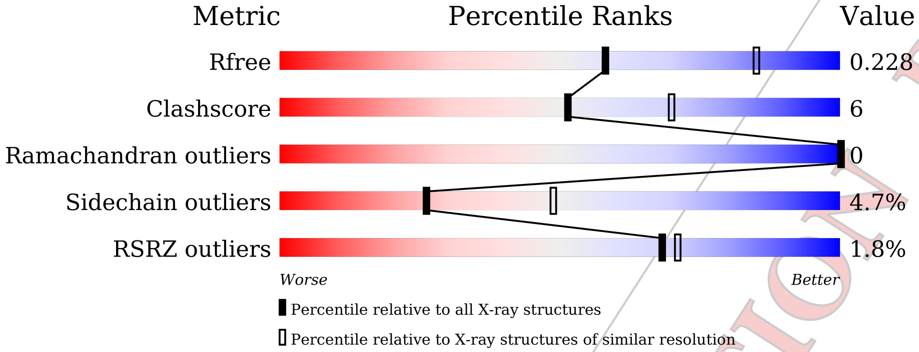

Metric	Whole archive (#Entries)	Similar resolution (#Entries, resolution range(Å))
R_{free}	130704	4661 (2.50-2.50)
Clashscore	141614	5346 (2.50-2.50)
Ramachandran outliers	138981	5231 (2.50-2.50)
Sidechain outliers	138945	5233 (2.50-2.50)
RSRZ outliers	127900	4559 (2.50-2.50)

The table below summarises the geometric issues observed across the polymeric chains and their fit to the electron density. The red, orange, yellow and green segments of the lower bar indicate the fraction of residues that contain outliers for >=3, 2, 1 and 0 types of geometric quality criteria respectively. A grey segment represents the fraction of residues that are not modelled. The numeric value for each fraction is indicated below the corresponding segment, with a dot representing fractions <=5% The upper red bar (where present) indicates the fraction of residues that have poor fit to the electron density. The numeric value is given above the bar.

Mol	Chain	Length	Quality of chain
1	A	341	% 86% 13% •
2	B	52	2% 88% 12%
3	C	338	% 85% 14% •
4	D	48	88% 12%
5	E	338	% 85% 12% •
6	F	54	2% 87% 11% •

Continued on next page...

PRELIMINARY VALIDATION REPORT

Continued from previous page...

Mol	Chain	Length	Quality of chain
7	G	339	% 82% 15% • •
8	H	51	6% 69% 31%
9	X	13	15% 69% 31%
10	I	10	30% 70% 30%
10	J	10	30% 50% 50%
10	K	10	20% 50% 50%

PRELIMINARY VALIDATION REPORT

2 Entry composition (i)

There are 11 unique types of molecules in this entry. The entry contains 13136 atoms, of which 0 are hydrogens and 0 are deuteriums.

In the tables below, the ZeroOcc column contains the number of atoms modelled with zero occupancy, the AltConf column contains the number of residues with at least one atom in alternate conformation and the Trace column contains the number of residues modelled with at most 2 atoms.

- Molecule 1 is a protein called human ribonucleic acid export 1.

Mol	Chain	Residues	Atoms					ZeroOcc	AltConf	Trace
			Total	C	N	O	S			
1	A	341	2704	1706	476	504	18	0	1	0

There are 10 discrepancies between the modelled and reference sequences:

Chain	Residue	Modelled	Actual	Comment	Reference
A	369	HIS	-	expression tag	UNP P78406
A	370	HIS	-	expression tag	UNP P78406
A	371	HIS	-	expression tag	UNP P78406
A	372	HIS	-	expression tag	UNP P78406
A	373	HIS	-	expression tag	UNP P78406
A	374	HIS	-	expression tag	UNP P78406
A	375	HIS	-	expression tag	UNP P78406
A	376	HIS	-	expression tag	UNP P78406
A	377	HIS	-	expression tag	UNP P78406
A	378	HIS	-	expression tag	UNP P78406

- Molecule 2 is a protein called human nucleoporin 98.

Mol	Chain	Residues	Atoms					ZeroOcc	AltConf	Trace
			Total	C	N	O	S			
2	B	52	412	252	70	87	3	0	0	0

There are 11 discrepancies between the modelled and reference sequences:

Chain	Residue	Modelled	Actual	Comment	Reference
B	147	MET	-	initiating methionine	UNP A0A1D8GZ55
B	148	HIS	-	expression tag	UNP A0A1D8GZ55
B	149	HIS	-	expression tag	UNP A0A1D8GZ55
B	150	HIS	-	expression tag	UNP A0A1D8GZ55
B	151	HIS	-	expression tag	UNP A0A1D8GZ55
B	152	HIS	-	expression tag	UNP A0A1D8GZ55

Continued on next page...

PRELIMINARY VALIDATION REPORT

Continued from previous page...

Chain	Residue	Modelled	Actual	Comment	Reference
B	153	HIS	-	expression tag	UNP A0A1D8GZ55
B	154	HIS	-	expression tag	UNP A0A1D8GZ55
B	155	HIS	-	expression tag	UNP A0A1D8GZ55
B	156	HIS	-	expression tag	UNP A0A1D8GZ55
B	157	HIS	-	expression tag	UNP A0A1D8GZ55

- Molecule 3 is a protein called human ribonucleic acid export 1.

Mol	Chain	Residues	Atoms					ZeroOcc	AltConf	Trace
			Total	C	N	O	S			
3	C	338	2674	1689	469	498	18	0	0	0

There are 10 discrepancies between the modelled and reference sequences:

Chain	Residue	Modelled	Actual	Comment	Reference
C	369	HIS	-	expression tag	UNP P78406
C	370	HIS	-	expression tag	UNP P78406
C	371	HIS	-	expression tag	UNP P78406
C	372	HIS	-	expression tag	UNP P78406
C	373	HIS	-	expression tag	UNP P78406
C	374	HIS	-	expression tag	UNP P78406
C	375	HIS	-	expression tag	UNP P78406
C	376	HIS	-	expression tag	UNP P78406
C	377	HIS	-	expression tag	UNP P78406
C	378	HIS	-	expression tag	UNP P78406

- Molecule 4 is a protein called human nucleoporin 98.

Mol	Chain	Residues	Atoms					ZeroOcc	AltConf	Trace
			Total	C	N	O	S			
4	D	48	384	235	66	81	2	0	0	0

There are 11 discrepancies between the modelled and reference sequences:

Chain	Residue	Modelled	Actual	Comment	Reference
D	147	MET	-	initiating methionine	UNP A0A1D8GZ55
D	148	HIS	-	expression tag	UNP A0A1D8GZ55
D	149	HIS	-	expression tag	UNP A0A1D8GZ55
D	150	HIS	-	expression tag	UNP A0A1D8GZ55
D	151	HIS	-	expression tag	UNP A0A1D8GZ55
D	152	HIS	-	expression tag	UNP A0A1D8GZ55
D	153	HIS	-	expression tag	UNP A0A1D8GZ55

Continued on next page...

PRELIMINARY VALIDATION REPORT

Continued from previous page...

Chain	Residue	Modelled	Actual	Comment	Reference
D	154	HIS	-	expression tag	UNP A0A1D8GZ55
D	155	HIS	-	expression tag	UNP A0A1D8GZ55
D	156	HIS	-	expression tag	UNP A0A1D8GZ55
D	157	HIS	-	expression tag	UNP A0A1D8GZ55

- Molecule 5 is a protein called human ribonucleic acid export 1.

Mol	Chain	Residues	Atoms					ZeroOcc	AltConf	Trace
			Total	C	N	O	S			
5	E	338	2674	1689	468	499	18	0	0	0

There are 10 discrepancies between the modelled and reference sequences:

Chain	Residue	Modelled	Actual	Comment	Reference
E	369	HIS	-	expression tag	UNP P78406
E	370	HIS	-	expression tag	UNP P78406
E	371	HIS	-	expression tag	UNP P78406
E	372	HIS	-	expression tag	UNP P78406
E	373	HIS	-	expression tag	UNP P78406
E	374	HIS	-	expression tag	UNP P78406
E	375	HIS	-	expression tag	UNP P78406
E	376	HIS	-	expression tag	UNP P78406
E	377	HIS	-	expression tag	UNP P78406
E	378	HIS	-	expression tag	UNP P78406

- Molecule 6 is a protein called human nucleoporin 98.

Mol	Chain	Residues	Atoms					ZeroOcc	AltConf	Trace
			Total	C	N	O	S			
6	F	54	423	259	72	89	3	0	0	0

There are 11 discrepancies between the modelled and reference sequences:

Chain	Residue	Modelled	Actual	Comment	Reference
F	147	MET	-	initiating methionine	UNP A0A1D8GZ55
F	148	HIS	-	expression tag	UNP A0A1D8GZ55
F	149	HIS	-	expression tag	UNP A0A1D8GZ55
F	150	HIS	-	expression tag	UNP A0A1D8GZ55
F	151	HIS	-	expression tag	UNP A0A1D8GZ55
F	152	HIS	-	expression tag	UNP A0A1D8GZ55
F	153	HIS	-	expression tag	UNP A0A1D8GZ55
F	154	HIS	-	expression tag	UNP A0A1D8GZ55

Continued on next page...

PRELIMINARY VALIDATION REPORT

Continued from previous page...

Chain	Residue	Modelled	Actual	Comment	Reference
F	155	HIS	-	expression tag	UNP A0A1D8GZ55
F	156	HIS	-	expression tag	UNP A0A1D8GZ55
F	157	HIS	-	expression tag	UNP A0A1D8GZ55

- Molecule 7 is a protein called human ribonucleic acid export 1.

Mol	Chain	Residues	Atoms					ZeroOcc	AltConf	Trace
			Total	C	N	O	S			
7	G	339	2686	1697	470	501	18	0	1	0

There are 10 discrepancies between the modelled and reference sequences:

Chain	Residue	Modelled	Actual	Comment	Reference
G	369	HIS	-	expression tag	UNP P78406
G	370	HIS	-	expression tag	UNP P78406
G	371	HIS	-	expression tag	UNP P78406
G	372	HIS	-	expression tag	UNP P78406
G	373	HIS	-	expression tag	UNP P78406
G	374	HIS	-	expression tag	UNP P78406
G	375	HIS	-	expression tag	UNP P78406
G	376	HIS	-	expression tag	UNP P78406
G	377	HIS	-	expression tag	UNP P78406
G	378	HIS	-	expression tag	UNP P78406

- Molecule 8 is a protein called human nucleoporin 98.

Mol	Chain	Residues	Atoms					ZeroOcc	AltConf	Trace
			Total	C	N	O	S			
8	H	51	405	247	69	86	3	0	0	0

There are 11 discrepancies between the modelled and reference sequences:

Chain	Residue	Modelled	Actual	Comment	Reference
H	147	MET	-	initiating methionine	UNP A0A1D8GZ55
H	148	HIS	-	expression tag	UNP A0A1D8GZ55
H	149	HIS	-	expression tag	UNP A0A1D8GZ55
H	150	HIS	-	expression tag	UNP A0A1D8GZ55
H	151	HIS	-	expression tag	UNP A0A1D8GZ55
H	152	HIS	-	expression tag	UNP A0A1D8GZ55
H	153	HIS	-	expression tag	UNP A0A1D8GZ55
H	154	HIS	-	expression tag	UNP A0A1D8GZ55
H	155	HIS	-	expression tag	UNP A0A1D8GZ55

Continued on next page...

PRELIMINARY VALIDATION REPORT

Continued from previous page...

Chain	Residue	Modelled	Actual	Comment	Reference
H	156	HIS	-	expression tag	UNP A0A1D8GZ55
H	157	HIS	-	expression tag	UNP A0A1D8GZ55

- Molecule 9 is a protein called ORF6 protein.

Mol	Chain	Residues	Atoms					ZeroOcc	AltConf	Trace
			Total	C	N	O	S			
9	X	13	109	66	13	29	1	0	0	0

- Molecule 10 is a protein called ORF6 protein.

Mol	Chain	Residues	Atoms					ZeroOcc	AltConf	Trace
			Total	C	N	O	S			
10	I	10	86	52	10	23	1	0	0	0
10	J	10	86	52	10	23	1	0	0	0
10	K	10	86	52	10	23	1	0	0	0

- Molecule 11 is water.

Mol	Chain	Residues	Atoms		ZeroOcc	AltConf
			Total	O		
11	S	407	407	407	0	0

PRELIMINARY VALIDATION REPORT

3 Residue-property plots (i)

These plots are drawn for all protein, RNA, DNA and oligosaccharide chains in the entry. The first graphic for a chain summarises the proportions of the various outlier classes displayed in the second graphic. The second graphic shows the sequence view annotated by issues in geometry and electron density. Residues are color-coded according to the number of geometric quality criteria for which they contain at least one outlier: green = 0, yellow = 1, orange = 2 and red = 3 or more. A red dot above a residue indicates a poor fit to the electron density (RSRZ > 2). Stretches of 2 or more consecutive residues without any outlier are shown as a green connector. Residues present in the sample, but not in the model, are shown in grey.

- Molecule 1: human ribonucleic acid export 1

Chain A:

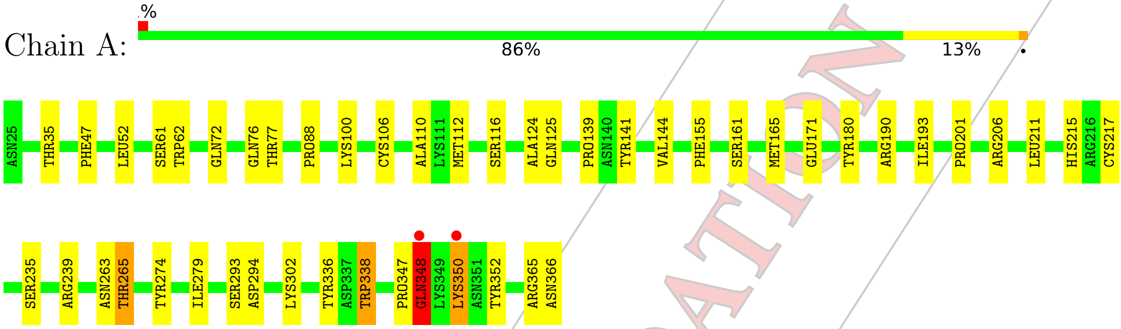

- Molecule 2: human nucleoporin 98

Chain B:

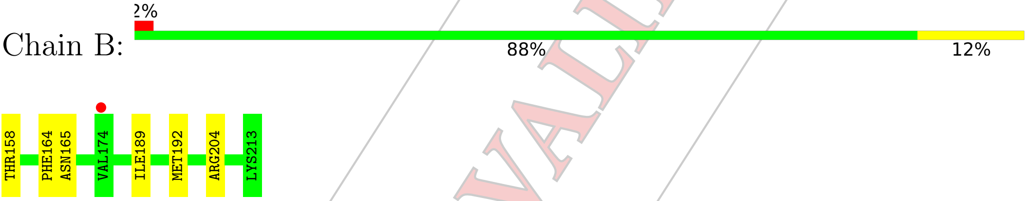

- Molecule 3: human ribonucleic acid export 1

Chain C:

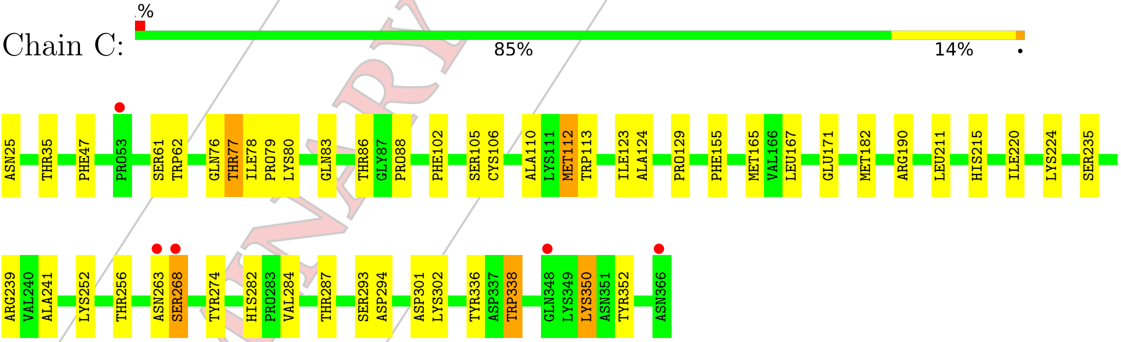

- Molecule 4: human nucleoporin 98

Chain D:

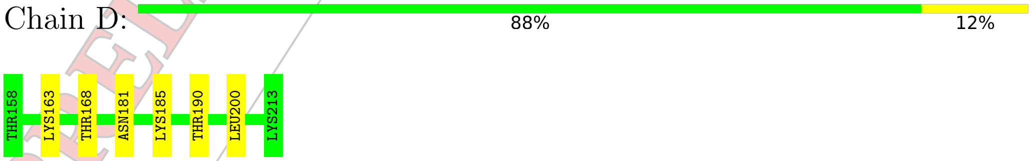

PRELIMINARY VALIDATION REPORT

- Molecule 5: human ribonucleic acid export 1

Chain E:

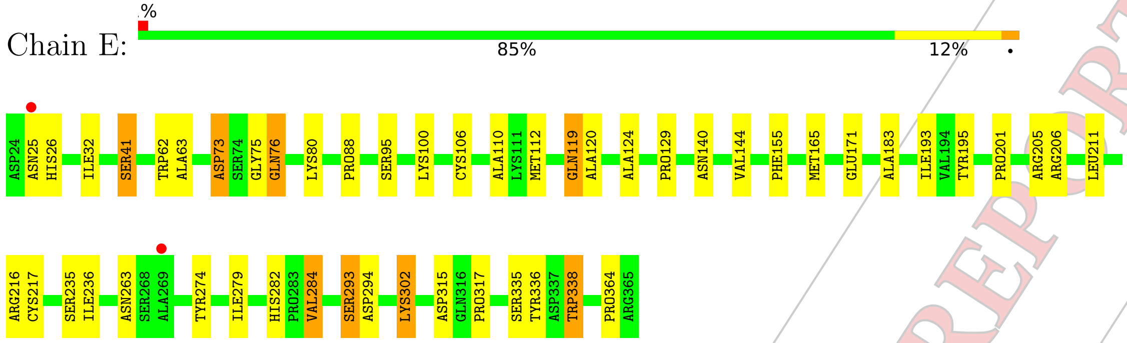

- Molecule 6: human nucleoporin 98

Chain F:

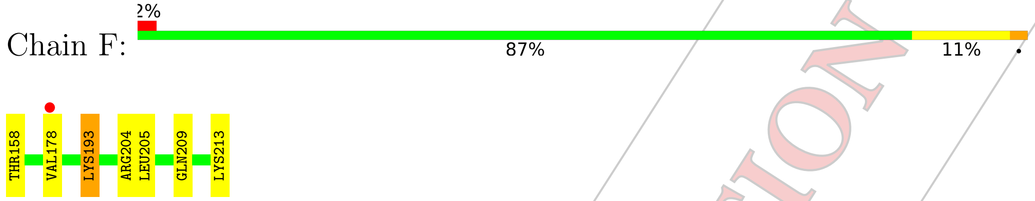

- Molecule 7: human ribonucleic acid export 1

Chain G:

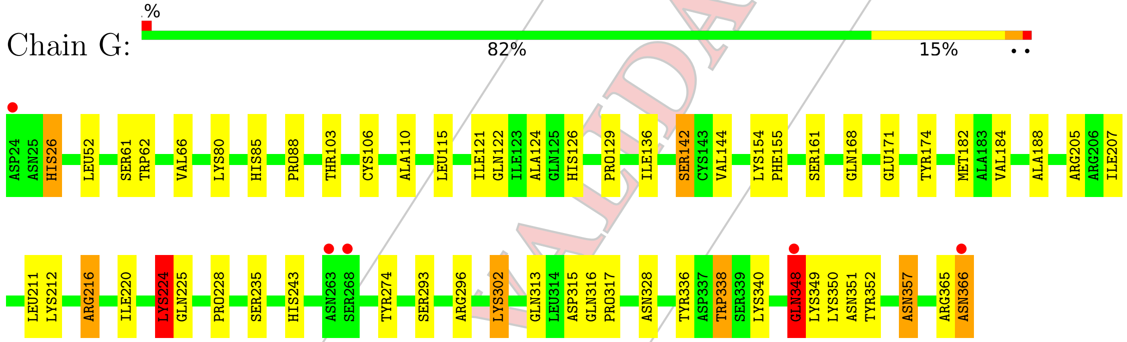

- Molecule 8: human nucleoporin 98

Chain H:

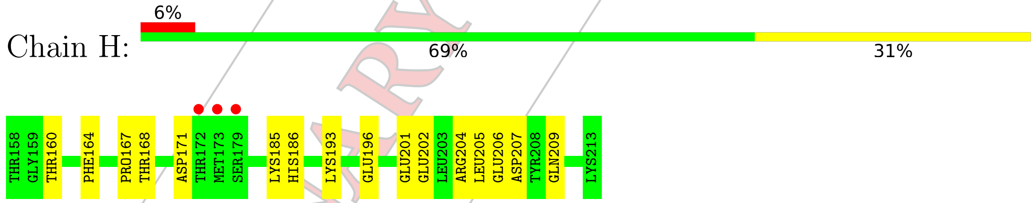

- Molecule 9: ORF6 protein

Chain X:

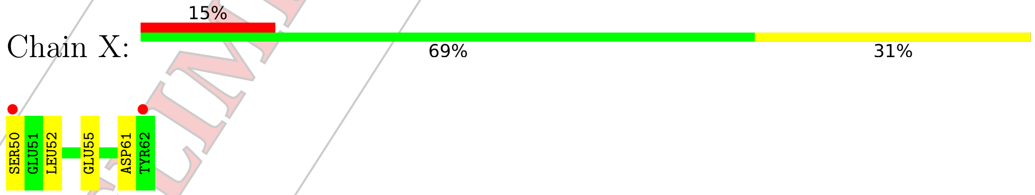

- Molecule 10: ORF6 protein

Chain I:

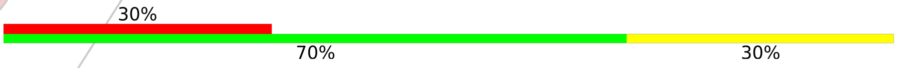

PRELIMINARY VALIDATION REPORT

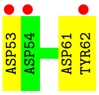

• Molecule 10: ORF6 protein

Chain J:

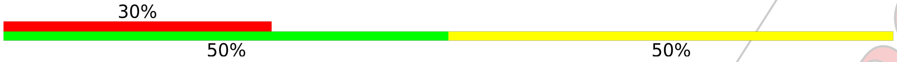

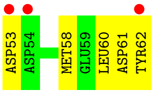

• Molecule 10: ORF6 protein

Chain K:

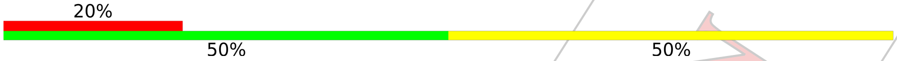

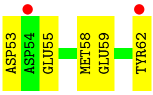

PRELIMINARY VALIDATION REPORT

4 Data and refinement statistics (i)

Property	Value	Source
Space group	C 1 2 1	Depositor
Cell constants<br>a, b, c, α, β, γ	149.61Å 103.30Å 134.94Å<br>90.00° 95.96° 90.00°	Depositor
Resolution (Å)	47.44 – 2.49<br>47.44 – 2.49	Depositor<br>EDS
% Data completeness<br>(in resolution range)	97.9 (47.44-2.49)<br>87.3 (47.44-2.49)	Depositor<br>EDS
R_{merge}	0.17	Depositor
R_{sym}	(Not available)	Depositor
$< I/\sigma(I) >$ [1]	0.59 (at 2.48Å)	Xtriage
Refinement program	PHENIX (1.19.2_4158: ???)	Depositor
R, R_{free}	0.186 , 0.230<br>0.185 , 0.228	Depositor<br>DCC
R_{free} test set	1963 reflections (2.79%)	wwPDB-VP
Wilson B-factor (Å^2)	34.5	Xtriage
Anisotropy	0.430	Xtriage
Bulk solvent k_{sol}(e/Å^3), B_{sol}(Å^2)	0.33 , 38.2	EDS
L-test for twinning[2]	$< \|L\| > = 0.49$, $< L^2 > = 0.32$	Xtriage
Estimated twinning fraction	No twinning to report.	Xtriage
F_o,F_c correlation	0.94	EDS
Total number of atoms	13136	wwPDB-VP
Average B, all atoms (Å^2)	45.0	wwPDB-VP

Xtriage's analysis on translational NCS is as follows: *The largest off-origin peak in the Patterson function is 3.58% of the height of the origin peak. No significant pseudotranslation is detected.*

[1] Intensities estimated from amplitudes.

[2] Theoretical values of $< |L| >$, $< L^2 >$ for acentric reflections are 0.5, 0.333 respectively for untwinned datasets, and 0.375, 0.2 for perfectly twinned datasets.

PRELIMINARY VALIDATION REPORT

5 Model quality (i)

5.1 Standard geometry (i)

The Z score for a bond length (or angle) is the number of standard deviations the observed value is removed from the expected value. A bond length (or angle) with $|Z| > 5$ is considered an outlier worth inspection. RMSZ is the root-mean-square of all Z scores of the bond lengths (or angles).

Mol	Chain	Bond lengths		Bond angles	
		RMSZ	#|Z| >5	RMSZ	#|Z| >5
1	A	0.51	1/2784 (0.0%)	0.80	5/3784 (0.1%)
2	B	0.41	0/416	0.65	0/557
3	C	0.44	0/2751	0.67	2/3739 (0.1%)
4	D	0.39	0/388	0.61	0/519
5	E	0.44	0/2751	0.66	1/3739 (0.0%)
6	F	0.46	0/427	0.63	0/572
7	G	0.43	0/2766	0.72	4/3760 (0.1%)
8	H	0.43	0/409	0.62	0/547
9	X	0.44	0/110	0.69	0/148
10	I	0.44	0/87	0.62	0/117
10	J	0.41	0/87	0.75	0/117
10	K	0.45	0/87	0.61	0/117
All	All	0.45	1/13063 (0.0%)	0.70	12/17716 (0.1%)

Chiral center outliers are detected by calculating the chiral volume of a chiral center and verifying if the center is modelled as a planar moiety or with the opposite hand.A planarity outlier is detected by checking planarity of atoms in a peptide group, atoms in a mainchain group or atoms of a sidechain that are expected to be planar.

Mol	Chain	#Chirality outliers	#Planarity outliers
1	A	0	3
5	E	0	1
7	G	0	1
All	All	0	5

All (1) bond length outliers are listed below:

Mol	Chain	Res	Type	Atoms	Z	Observed(Å)	Ideal(Å)
1	A	350	LYS	CD-CE	7.85	1.70	1.51

All (12) bond angle outliers are listed below:

PRELIMINARY VALIDATION REPORT

Mol	Chain	Res	Type	Atoms	Z	Observed(°)	Ideal(°)
1	A	350	LYS	CD-CE-NZ	20.96	159.91	111.70
7	G	348	GLN	CA-CB-CG	11.43	138.55	113.40
3	C	268	SER	C-N-CA	10.66	148.35	121.70
7	G	348	GLN	CB-CA-C	-9.87	90.67	110.40
1	A	348	GLN	CA-CB-CG	8.57	132.26	113.40
7	G	348	GLN	N-CA-CB	8.06	125.11	110.60
1	A	348	GLN	N-CA-CB	6.32	121.98	110.60
5	E	76	GLN	CB-CA-C	5.88	122.15	110.40
1	A	350	LYS	CG-CD-CE	5.28	127.75	111.90
7	G	224	LYS	CA-CB-CG	5.25	124.95	113.40
1	A	348	GLN	CB-CA-C	-5.22	99.96	110.40
3	C	268	SER	CA-C-N	-5.06	106.08	117.20

There are no chirality outliers.

All (5) planarity outliers are listed below:

Mol	Chain	Res	Type	Group
1	A	347	PRO	Peptide
1	A	348	GLN	Sidechain,Peptide
5	E	76	GLN	Peptide
7	G	348	GLN	Sidechain

5.2 Too-close contacts (i)

In the following table, the Non-H and H(model) columns list the number of non-hydrogen atoms and hydrogen atoms in the chain respectively. The H(added) column lists the number of hydrogen atoms added and optimized by MolProbity. The Clashes column lists the number of clashes within the asymmetric unit, whereas Symm-Clashes lists symmetry-related clashes.

Mol	Chain	Non-H	H(model)	H(added)	Clashes	Symm-Clashes
1	A	2704	0	2601	28	1
2	B	412	0	406	8	0
3	C	2674	0	2568	26	0
4	D	384	0	376	5	0
5	E	2674	0	2566	25	1
6	F	423	0	418	4	0
7	G	2686	0	2581	39	1
8	H	405	0	397	9	0
9	X	109	0	87	4	0
10	I	86	0	65	1	0
10	J	86	0	65	1	1
10	K	86	0	65	2	0

Continued on next page...

PRELIMINARY VALIDATION REPORT

Continued from previous page...

Mol	Chain	Non-H	H(model)	H(added)	Clashes	Symm-Clashes
11	S	407	0	0	8	0
All	All	13136	0	12195	139	2

The all-atom clashscore is defined as the number of clashes found per 1000 atoms (including hydrogen atoms). The all-atom clashscore for this structure is 6.

All (139) close contacts within the same asymmetric unit are listed below, sorted by their clash magnitude.

Atom-1	Atom-2	Interatomic distance (Å)	Clash overlap (Å)
5:E:140:ASN:ND2	11:S:424:HOH:O	2.15	0.77
5:E:211:LEU:HD13	5:E:235:SER:HB3	1.68	0.76
8:H:168:THR:HG22	8:H:185:LYS:HD3	1.70	0.73
1:A:193:ILE:HG12	1:A:206[A]:ARG:HD3	1.69	0.73
7:G:357:ASN:ND2	11:S:418:HOH:O	2.21	0.72
2:B:158:THR:N	11:S:303:HOH:O	2.27	0.67
2:B:165:ASN:H	2:B:192:MET:HE2	1.60	0.65
3:C:211:LEU:HD13	3:C:235:SER:HB3	1.76	0.65
7:G:350:LYS:HG2	7:G:352:TYR:CZ	2.32	0.65
4:D:181:ASN:N	11:S:137:HOH:O	2.28	0.65
1:A:263:ASN:OD1	11:S:322:HOH:O	2.15	0.64
8:H:167:PRO:HD2	8:H:186:HIS:HB2	1.80	0.61
7:G:211:LEU:HD13	7:G:235:SER:HB3	1.82	0.61
7:G:182:MET:CE	7:G:228:PRO:HB2	2.31	0.61
7:G:366:ASN:O	7:G:366:ASN:ND2	2.35	0.60
3:C:88:PRO:HD2	3:C:106:CYS:HB2	1.82	0.60
3:C:182:MET:HE2	3:C:220:ILE:HG12	1.84	0.59
5:E:119:GLN:HA	5:E:119:GLN:OE1	2.02	0.59
6:F:193:LYS:H	6:F:193:LYS:CD	2.16	0.58
5:E:193:ILE:HG12	5:E:206:ARG:HG2	1.85	0.58
2:B:164:PHE:HA	2:B:192:MET:CE	2.34	0.57
3:C:106:CYS:HA	3:C:129:PRO:HB3	1.87	0.56
5:E:282:HIS:CE1	5:E:284:VAL:HG22	2.42	0.55
1:A:239:ARG:NH1	9:X:61:ASP:OD2	2.40	0.54
5:E:41:SER:OG	6:F:204:ARG:NH2	2.38	0.54
3:C:110:ALA:HB3	3:C:124:ALA:HB3	1.89	0.54
7:G:121:ILE:HD12	7:G:122:GLN:H	1.72	0.54
3:C:282:HIS:CE1	3:C:284:VAL:HB	2.44	0.53
3:C:282:HIS:HE1	3:C:284:VAL:HB	1.74	0.53
3:C:62:TRP:HA	3:C:88:PRO:HB3	1.91	0.53
3:C:79:PRO:HD2	9:X:52:LEU:HB2	1.91	0.53
1:A:350:LYS:HZ2	1:A:352:TYR:HE1	1.56	0.52

Continued on next page...

PRELIMINARY VALIDATION REPORT

Continued from previous page...

Atom-1	Atom-2	Interatomic distance (Å)	Clash overlap (Å)
5:E:110:ALA:HB3	5:E:124:ALA:HB3	1.91	0.52
7:G:26:HIS:H	7:G:26:HIS:CD2	2.28	0.52
8:H:205:LEU:O	8:H:209:GLN:HG3	2.09	0.52
5:E:274:TYR:CD1	5:E:294:ASP:HB3	2.45	0.52
7:G:182:MET:HE3	7:G:228:PRO:HB2	1.92	0.51
3:C:350:LYS:HB3	3:C:352:TYR:CE2	2.46	0.51
5:E:112:MET:O	5:E:120:ALA:HA	2.11	0.51
3:C:102:PHE:CE1	3:C:112:MET:HG3	2.46	0.50
7:G:216:ARG:NH2	8:H:201:GLU:OE2	2.44	0.50
1:A:211:LEU:HD13	1:A:235:SER:HB3	1.92	0.50
7:G:315:ASP:OD1	7:G:315:ASP:N	2.44	0.49
8:H:202:GLU:O	8:H:206:GLU:HG3	2.12	0.49
7:G:184[B]:VAL:HG23	7:G:220:ILE:HD11	1.95	0.49
2:B:164:PHE:HA	2:B:192:MET:HE1	1.93	0.49
3:C:190:ARG:HA	3:C:215:HIS:CE1	2.48	0.49
3:C:274:TYR:CD2	3:C:294:ASP:HB3	2.48	0.49
8:H:193:LYS:HE3	8:H:196:GLU:OE2	2.13	0.49
7:G:296:ARG:NH1	7:G:313:GLN:HB2	2.27	0.49
6:F:205:LEU:O	6:F:209:GLN:HG3	2.13	0.49
7:G:110:ALA:HB3	7:G:124:ALA:HB3	1.95	0.48
7:G:174:TYR:CZ	7:G:188:ALA:HB2	2.48	0.48
1:A:62:TRP:HA	1:A:88:PRO:HB3	1.94	0.48
5:E:293:SER:HA	5:E:317:PRO:HB3	1.94	0.48
1:A:190:ARG:HA	1:A:215:HIS:CE1	2.48	0.48
3:C:78:ILE:HG22	3:C:80:LYS:HD3	1.96	0.48
1:A:365:ARG:O	1:A:366:ASN:HB2	2.14	0.47
7:G:106:CYS:HA	7:G:129:PRO:HB3	1.96	0.47
3:C:165:MET:HE1	3:C:167:LEU:HD11	1.97	0.47
7:G:224:LYS:HB3	7:G:225:GLN:OE1	2.15	0.47
1:A:239:ARG:NH1	9:X:61:ASP:OD1	2.48	0.46
5:E:106:CYS:HA	5:E:129:PRO:HB3	1.97	0.46
3:C:76:GLN:NE2	9:X:55:GLU:OE1	2.45	0.46
4:D:168:THR:HG22	4:D:185:LYS:HD3	1.98	0.46
3:C:241:ALA:HA	3:C:256:THR:HG22	1.97	0.46
7:G:274:TYR:HB3	7:G:293:SER:HB2	1.97	0.46
7:G:316:GLN:CD	7:G:349:LYS:HG2	2.36	0.46
1:A:144:VAL:O	1:A:155:PHE:HA	2.15	0.46
2:B:164:PHE:HA	2:B:192:MET:HE2	1.97	0.46
5:E:32:ILE:HG21	5:E:75:GLY:O	2.16	0.46
7:G:293:SER:HA	7:G:317:PRO:HB3	1.97	0.46
1:A:190:ARG:HD2	1:A:211:LEU:O	2.15	0.45

Continued on next page...

PRELIMINARY VALIDATION REPORT

Continued from previous page...

Atom-1	Atom-2	Interatomic distance (Å)	Clash overlap (Å)
5:E:62:TRP:HA	5:E:88:PRO:HB3	1.98	0.45
5:E:165:MET:HE1	5:E:201:PRO:HD2	1.97	0.45
1:A:110:ALA:HB3	1:A:124:ALA:HB3	1.99	0.45
7:G:136:ILE:O	7:G:142:SER:HA	2.15	0.45
1:A:35:THR:HG23	1:A:77:THR:OG1	2.16	0.45
1:A:165:MET:HE1	1:A:201:PRO:HD2	1.99	0.45
1:A:350:LYS:NZ	1:A:352:TYR:HE1	2.15	0.45
7:G:144:VAL:O	7:G:155:PHE:HA	2.17	0.45
4:D:163:LYS:HE2	6:F:213:LYS:O	2.17	0.45
1:A:141:TYR:OH	1:A:201:PRO:HD3	2.17	0.44
2:B:189:ILE:HG13	2:B:192:MET:HE3	2.00	0.44
7:G:85:HIS:NE2	7:G:103:THR:OG1	2.41	0.44
4:D:190:THR:HG23	4:D:200:LEU:HD23	1.98	0.44
1:A:336:TYR:CE2	1:A:338:TRP:HA	2.53	0.44
1:A:100:LYS:HD2	1:A:112:MET:SD	2.57	0.44
3:C:336:TYR:CE2	3:C:338:TRP:HA	2.52	0.44
1:A:52:LEU:HD12	1:A:52:LEU:HA	1.79	0.44
1:A:338:TRP:CD2	2:B:204:ARG:HG3	2.53	0.44
3:C:80:LYS:HA	3:C:80:LYS:HD2	1.66	0.44
5:E:144:VAL:O	5:E:155:PHE:HA	2.18	0.44
1:A:274:TYR:CD2	1:A:294:ASP:HB3	2.53	0.43
10:J:58:MET:O	10:J:60:LEU:HD22	2.17	0.43
3:C:155:PHE:CD1	3:C:165:MET:HB3	2.53	0.43
7:G:80:LYS:HD3	7:G:80:LYS:HA	1.73	0.43
7:G:207:ILE:HD13	7:G:243:HIS:NE2	2.33	0.43
5:E:336:TYR:CE2	5:E:338:TRP:HA	2.53	0.43
7:G:350:LYS:HG3	7:G:351:ASN:N	2.32	0.43
5:E:302:LYS:HG3	11:S:183:HOH:O	2.18	0.43
7:G:336:TYR:CE2	7:G:338:TRP:HA	2.53	0.43
7:G:182:MET:HE2	7:G:228:PRO:HB2	2.00	0.43
1:A:88:PRO:HD2	1:A:106:CYS:HB2	2.01	0.43
7:G:365:ARG:HD2	10:K:55:GLU:HB2	2.01	0.43
3:C:287:THR:HG22	3:C:301:ASP:HA	2.00	0.43
7:G:302:LYS:HG3	11:S:4:HOH:O	2.18	0.43
8:H:160:THR:OG1	8:H:207:ASP:OD2	2.35	0.43
3:C:83:GLN:HG2	3:C:113:TRP:CE2	2.55	0.42
5:E:41:SER:HB3	5:E:335:SER:HB3	2.02	0.42
7:G:62:TRP:HA	7:G:88:PRO:HB3	2.00	0.42
7:G:115:LEU:HD23	7:G:115:LEU:HA	1.89	0.42
1:A:263:ASN:C	1:A:265:THR:HG23	2.40	0.42
5:E:88:PRO:HD2	5:E:106:CYS:HB2	2.00	0.42

Continued on next page...

PRELIMINARY VALIDATION REPORT

Continued from previous page...

Atom-1	Atom-2	Interatomic distance (Å)	Clash overlap (Å)
7:G:52:LEU:HD12	7:G:52:LEU:HA	1.78	0.42
3:C:112:MET:HB2	3:C:123:ILE:HG21	2.01	0.42
5:E:183:ALA:HB3	5:E:195:TYR:HB2	2.01	0.42
1:A:72:GLN:OE1	1:A:76:GLN:NE2	2.53	0.41
7:G:62:TRP:CZ3	8:H:204:ARG:HG2	2.55	0.41
7:G:126:HIS:CE1	7:G:154:LYS:HD2	2.55	0.41
1:A:217:CYS:SG	1:A:279:ILE:HG13	2.61	0.41
4:D:163:LYS:HA	4:D:163:LYS:HD3	1.82	0.41
1:A:165:MET:HE1	1:A:201:PRO:CD	2.51	0.41
5:E:73:ASP:C	5:E:75:GLY:H	2.23	0.41
3:C:35:THR:HG23	3:C:77:THR:OG1	2.20	0.41
5:E:95:SER:HB3	5:E:100:LYS:HB2	2.02	0.41
1:A:139:PRO:HD2	1:A:180:TYR:CZ	2.56	0.41
2:B:189:ILE:HG13	2:B:192:MET:CE	2.51	0.41
5:E:26:HIS:HA	5:E:315:ASP:OD2	2.21	0.41
5:E:217:CYS:SG	5:E:279:ILE:HG13	2.61	0.41
7:G:328:ASN:O	7:G:357:ASN:HA	2.21	0.41
5:E:216:ARG:HD2	5:E:236:ILE:HG22	2.03	0.40
3:C:190:ARG:HD2	3:C:211:LEU:O	2.21	0.40
7:G:26:HIS:H	7:G:26:HIS:HD2	1.69	0.40
7:G:302:LYS:HA	10:K:58:MET:HE1	2.03	0.40
1:A:100:LYS:HD2	1:A:112:MET:CG	2.51	0.40
7:G:340:LYS:HD3	8:H:164:PHE:CE2	2.57	0.40
3:C:239:ARG:NH1	10:I:61:ASP:OD1	2.54	0.40
7:G:168:GLN:NE2	11:S:457:HOH:O	2.55	0.40

All (2) symmetry-related close contacts are listed below. The label for Atom-2 includes the symmetry operator and encoded unit-cell translations to be applied.

Atom-1	Atom-2	Interatomic distance (Å)	Clash overlap (Å)
5:E:63:ALA:O	7:G:348:GLN:NE2[2_556]	1.95	0.25
1:A:190:ARG:NH2	10:J:62:TYR:OH[3_455]	2.15	0.05

5.3 Torsion angles (i)

5.3.1 Protein backbone (i)

In the following table, the Percentiles column shows the percent Ramachandran outliers of the chain as a percentile score with respect to all X-ray entries followed by that with respect to entries of similar resolution.

PRELIMINARY VALIDATION REPORT

The Analysed column shows the number of residues for which the backbone conformation was analysed, and the total number of residues.

Mol	Chain	Analysed	Favoured	Allowed	Outliers	Percentiles	
1	A	338/341 (99%)	330 (98%)	8 (2%)	0	100	100
2	B	48/52 (92%)	47 (98%)	1 (2%)	0	100	100
3	C	334/338 (99%)	327 (98%)	7 (2%)	0	100	100
4	D	44/48 (92%)	43 (98%)	1 (2%)	0	100	100
5	E	334/338 (99%)	327 (98%)	7 (2%)	0	100	100
6	F	50/54 (93%)	49 (98%)	1 (2%)	0	100	100
7	G	336/339 (99%)	327 (97%)	9 (3%)	0	100	100
8	H	47/51 (92%)	46 (98%)	1 (2%)	0	100	100
9	X	11/13 (85%)	10 (91%)	1 (9%)	0	100	100
10	I	8/10 (80%)	8 (100%)	0	0	100	100
10	J	8/10 (80%)	8 (100%)	0	0	100	100
10	K	8/10 (80%)	8 (100%)	0	0	100	100
All	All	1566/1604 (98%)	1530 (98%)	36 (2%)	0	100	100

There are no Ramachandran outliers to report.

5.3.2 Protein sidechains (i)

In the following table, the Percentiles column shows the percent sidechain outliers of the chain as a percentile score with respect to all X-ray entries followed by that with respect to entries of similar resolution.

The Analysed column shows the number of residues for which the sidechain conformation was analysed, and the total number of residues.

Mol	Chain	Analysed	Rotameric	Outliers	Percentiles	
1	A	298/297 (100%)	287 (96%)	11 (4%)	34	60
2	B	48/48 (100%)	48 (100%)	0	100	100
3	C	294/294 (100%)	278 (95%)	16 (5%)	22	42
4	D	44/44 (100%)	44 (100%)	0	100	100
5	E	294/294 (100%)	281 (96%)	13 (4%)	28	52
6	F	49/49 (100%)	46 (94%)	3 (6%)	18	36
7	G	296/295 (100%)	281 (95%)	15 (5%)	24	45

Continued on next page...

PRELIMINARY VALIDATION REPORT

Continued from previous page...

Mol	Chain	Analysed	Rotameric	Outliers	Percentiles	
8	H	47/47 (100%)	46 (98%)	1 (2%)	53	78
9	X	13/13 (100%)	12 (92%)	1 (8%)	13	25
10	I	10/10 (100%)	8 (80%)	2 (20%)	1	2
10	J	10/10 (100%)	8 (80%)	2 (20%)	1	2
10	K	10/10 (100%)	7 (70%)	3 (30%)	0	0
All	All	1413/1411 (100%)	1346 (95%)	67 (5%)	26	49

All (67) residues with a non-rotameric sidechain are listed below:

Mol	Chain	Res	Type
1	A	47	PHE
1	A	61	SER
1	A	116	SER
1	A	125	GLN
1	A	161	SER
1	A	171	GLU
1	A	265	THR
1	A	293	SER
1	A	302	LYS
1	A	338	TRP
1	A	348	GLN
3	C	25	ASN
3	C	47	PHE
3	C	61	SER
3	C	77	THR
3	C	86	THR
3	C	105	SER
3	C	112	MET
3	C	171	GLU
3	C	224	LYS
3	C	252	LYS
3	C	263	ASN
3	C	268	SER
3	C	293	SER
3	C	302	LYS
3	C	338	TRP
3	C	350	LYS
5	E	25	ASN
5	E	41	SER
5	E	73	ASP

Continued on next page...

PRELIMINARY VALIDATION REPORT

Continued from previous page...

Mol	Chain	Res	Type
5	E	80	LYS
5	E	119	GLN
5	E	171	GLU
5	E	205	ARG
5	E	263	ASN
5	E	284	VAL
5	E	293	SER
5	E	302	LYS
5	E	338	TRP
5	E	364	PRO
6	F	158	THR
6	F	178	VAL
6	F	193	LYS
7	G	26	HIS
7	G	61	SER
7	G	66	VAL
7	G	142	SER
7	G	161	SER
7	G	171	GLU
7	G	205	ARG
7	G	212	LYS
7	G	216	ARG
7	G	224	LYS
7	G	302	LYS
7	G	338	TRP
7	G	348	GLN
7	G	357	ASN
7	G	366	ASN
8	H	171	ASP
9	X	50	SER
10	I	53	ASP
10	I	62	TYR
10	J	53	ASP
10	J	61	ASP
10	K	53	ASP
10	K	59	GLU
10	K	62	TYR

Sometimes sidechains can be flipped to improve hydrogen bonding and reduce clashes. All (11) such sidechains are listed below:

Mol	Chain	Res	Type
1	A	25	ASN

Continued on next page...

PRELIMINARY VALIDATION REPORT

Continued from previous page...

Mol	Chain	Res	Type
1	A	76	GLN
3	C	348	GLN
4	D	186	HIS
4	D	211	ASN
5	E	76	GLN
5	E	162	ASN
5	E	263	ASN
5	E	285	HIS
7	G	316	GLN
8	H	187	GLN

5.3.3 RNA (i)

There are no RNA molecules in this entry.

5.4 Non-standard residues in protein, DNA, RNA chains (i)

There are no non-standard protein/DNA/RNA residues in this entry.

5.5 Carbohydrates (i)

There are no monosaccharides in this entry.

5.6 Ligand geometry (i)

There are no ligands in this entry.

5.7 Other polymers (i)

There are no such residues in this entry.

5.8 Polymer linkage issues (i)

The following chains have linkage breaks:

Mol	Chain	Number of breaks
7	G	1
3	C	1

Continued on next page...

PRELIMINARY VALIDATION REPORT

Continued from previous page...

Mol	Chain	Number of breaks
5	E	1
6	F	1
2	B	1
8	H	1
4	D	1
1	A	1

All chain breaks are listed below:

Model	Chain	Residue-1	Atom-1	Residue-2	Atom-2	Distance (Å)
1	G	263:ASN	C	268:SER	N	9.21
1	C	263:ASN	C	268:SER	N	7.82
1	E	263:ASN	C	268:SER	N	7.25
1	F	174:VAL	C	177:GLY	N	6.19
1	B	174:VAL	C	179:SER	N	5.29
1	H	173:MET	C	179:SER	N	5.18
1	D	172:THR	C	181:ASN	N	4.98
1	A	263:ASN	C	265:THR	N	4.74

PRELIMINARY VALIDATION REPORT

6 Fit of model and data (i)

6.1 Protein, DNA and RNA chains (i)

In the following table, the column labelled '#RSRZ> 2' contains the number (and percentage) of RSRZ outliers, followed by percent RSRZ outliers for the chain as percentile scores relative to all X-ray entries and entries of similar resolution. The OWAB column contains the minimum, median, 95th percentile and maximum values of the occupancy-weighted average B-factor per residue. The column labelled 'Q< 0.9' lists the number of (and percentage) of residues with an average occupancy less than 0.9.

Mol	Chain	Analysed	<RSRZ>	#RSRZ>2			OWAB(Å^2)	Q<0.9
1	A	341/341 (100%)	-0.40	2 (0%)	89	90	25, 35, 56, 101	0
2	B	52/52 (100%)	-0.03	1 (1%)	66	69	31, 43, 84, 92	0
3	C	338/338 (100%)	-0.09	5 (1%)	73	75	30, 45, 66, 102	0
4	D	48/48 (100%)	-0.04	0	100	100	37, 52, 84, 89	0
5	E	338/338 (100%)	-0.20	2 (0%)	89	90	28, 40, 64, 113	0
6	F	54/54 (100%)	-0.12	1 (1%)	66	69	33, 46, 70, 86	0
7	G	339/339 (100%)	-0.24	5 (1%)	73	75	28, 44, 69, 108	0
8	H	51/51 (100%)	0.20	3 (5%)	22	23	41, 60, 100, 108	0
9	X	13/13 (100%)	0.85	2 (15%)	2	1	43, 56, 94, 101	0
10	I	10/10 (100%)	0.91	3 (30%)	0	0	48, 62, 86, 90	0
10	J	10/10 (100%)	0.78	3 (30%)	0	0	43, 63, 92, 96	0
10	K	10/10 (100%)	0.78	2 (20%)	1	0	48, 63, 88, 91	0
All	All	1604/1604 (100%)	-0.18	29 (1%)	68	71	25, 43, 71, 113	0

All (29) RSRZ outliers are listed below:

Mol	Chain	Res	Type	RSRZ
9	X	50	SER	4.8
3	C	366	ASN	3.6
3	C	263	ASN	3.4
1	A	348	GLN	3.4
8	H	172	THR	3.3
3	C	268	SER	3.3
6	F	178	VAL	3.2
7	G	263	ASN	3.2
5	E	25	ASN	3.1
7	G	348	GLN	3.1
8	H	173	MET	3.1

Continued on next page...

PRELIMINARY VALIDATION REPORT

Continued from previous page...

Mol	Chain	Res	Type	RSRZ
9	X	62	TYR	3.0
10	I	62	TYR	3.0
10	J	54	ASP	2.8
3	C	348	GLN	2.7
5	E	269	ALA	2.7
7	G	268	SER	2.6
7	G	24	ASP	2.6
10	K	54	ASP	2.6
10	I	54	ASP	2.5
1	A	350	LYS	2.4
10	J	53	ASP	2.4
10	I	53	ASP	2.4
8	H	179	SER	2.3
3	C	53	PRO	2.2
10	J	62	TYR	2.2
2	B	174	VAL	2.2
7	G	366	ASN	2.1
10	K	62	TYR	2.0

6.2 Non-standard residues in protein, DNA, RNA chains (i)

There are no non-standard protein/DNA/RNA residues in this entry.

6.3 Carbohydrates (i)

There are no monosaccharides in this entry.

6.4 Ligands (i)

There are no ligands in this entry.

6.5 Other polymers (i)

There are no such residues in this entry.

PRELIMINARY VALIDATION REPORT
